# Computational drill down on FGF1-heparin interactions through methodological evaluation

**DOI:** 10.1007/s10719-016-9745-4

**Published:** 2016-11-17

**Authors:** Sándor Babik, Sergey A. Samsonov, M. Teresa Pisabarro

**Affiliations:** Structural Bioinformatics, BIOTEC TU Dresden, Dresden, 01307 Germany

**Keywords:** FGF, GAG, Heparin, Docking, Molecular dynamics, MM-GBSA, Site-mapping

## Abstract

Glycosaminoglycans (GAGs) exhibit a key role in cellular communication processes through interactions with target proteins of the extracellular matrix (ECM). The sandwich-like interaction established between Fibroblast growth factor (FGF) and heparin (HE) represents quite a peculiar protein-GAG-protein system, which has been both structurally and functionally intensively studied. The molecular recognition characteristics of this system have been exploited in various computational studies in order to deepen understanding of GAG-protein interactions. Here, we drill down on the interactions established in this peculiar macromolecular complex by analyzing the applicability of docking techniques and molecular dynamics (MD)-based approaches, and we dissect the molecular recognition properties exhibited by FGF towards a series of HE derivatives. We examine the sensitivity of MM-GBSA free energy calculations in terms of receptor conformational space sampling and changes in the ligand structures. Furthermore, we investigate its predictive power in combination with other computational methods, namely the well-established Autodock3 (AD3) and dynamic molecular docking (DMD), a targeted MD-based docking method specifically developed to account for flexibility and solvent in computer simulations of protein-GAG systems. Our results show that a site-mapping approach can be effectively combined with AD3 and DMD calculations to accurately reproduce available experimental data and, furthermore, to determine specific GAG recognition patterns. This study deepens our understanding of the applicability of available theoretical approaches to the investigation of molecular recognition in protein-GAG systems.

**Electronic supplementary material:**

The online version of this article (doi:10.1007/s10719-016-9745-4) contains supplementary material, which is available to authorized users.

## Introduction

Glycosaminoglycans (GAGs) represent a class of anionic linear polysaccharides made up of repetitive disaccharide units containing an amino sugar and an uronic acid [1]. In the extracellular matrix (ECM), the binding of GAGs to growth factors and chemokines [2, 3] makes them key participants in cellular communication processes [4]. Protein-GAG interactions represent very challenging systems for computational studies due to their intrinsic molecular properties. First of all, their highly charged nature leads to electrostatics-driven [5] and abundant solvent-mediated interactions, which require proper computational treatment [6]. Along with that, GAGs usually bind proteins at solvent-exposed and spatially close but sequentially not necessarily successive positively charged amino acid patches [7, 8] and, due to the fact that these amino acids (*i.e.* Lys, Arg) possess long and flexible side-chains, it makes protein flexibility a crucial aspect to take into account in the analysis of GAG binding. When applying the Molecular Mechanic Generalized Born Surface Area (MM-GBSA) methodology for calculating free energy of binding in protein-GAG systems, which has been widely used for such systems since the pioneering studies of PECAM-1 and annexin 2 [9], even slight movements related to the thermal fluctuations of the charged groups lead to dramatic changes in the interaction energy, which results in high standard deviations for the calculated values and, therefore, in the difficulty for revealing significant differences between them [10, 11]. It was also shown that GAG binding poses obtained from crystal structures and also by molecular docking could both be altered during MD simulations due to the changes in protein secondary structure or newly established water-mediated interactions [9]. Furthermore, the periodic nature of GAG polymers makes it nontrivial to computationally distinguish their distinct binding poses [12], which increases the possibility for multipose binding [13, 14]. Besides, information about GAG puckering conformations, which is a crucial aspect for their binding specificity [15] and bioactivity [16], and therefore key for modeling GAG-protein interactions [17], is rarely experimentally available *a priori*. Unfortunately, conventional molecular dynamics (MD) approaches cannot easily deal with this due to the μs time scales needed to be able to observe equilibrium between different ring puckering [18, 19]. By virtue of all these challenges, and opposite to other biomacromolecular systems, standard computational pipelines for treatment of protein-GAG interactions are not yet fully established but rather currently intensively pursued. The development of carbohydrate-related force fields and new scoring functions in the last decade [20–23] has made possible the theoretical analysis of protein-GAG interactions in a large number of representative systems [10, 11, 24–34].

**Fig. 1:**
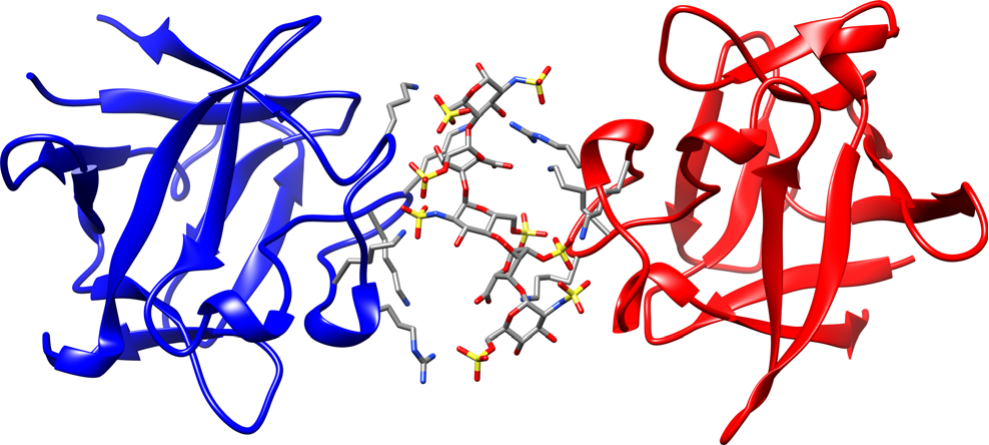
Detail of the crystallographic complex FGF1-HE dp5-FGF1 (PDB ID 1AMX, 3.0 Å) showing each protein chain (FGF_A_ and FGF_B_) in red and blue cartoon and the HE dp5 ligand in sticks and coloured by atom. The side chains of the protein positively-charged residues in the binding interface are displayed in ﻿grey sticks

Perhaps one of the most widely studied protein-GAG systems is the fibroblast growth factor (FGF)-heparin (FGF-HE) complex. HE is a GAG made up of 6-O-sulfated N-sulfated glucosamine (GlcNS) and 2-O-sulfated iduronic acid (IdoA2S) repetitive units. The mammalian FGF protein family is comprised of 18 members, which are involved in a wide range of functions from development and regenerative processes to metabolism and tissue homeostasis [35–37]. FGFs carry out their biological functions by binding and dimerizing FGF receptor-tyrosine kinases (FGFRs) [38, 39]. The core homology domain of FGFs consists of about 120 amino acids arranged in a β-trefoil fold made up of 12 antiparallel β-strands [40]. Receptor dimerization and thereby the FGF signaling pathway require the presence of HE or HE derivatives [41–44]. Indeed, it has been shown that FGF1 (human recombinant acidic Fibroblast Growth Factor) forms a 2:1 sandwich-like dimer structure with HE, which does not present any direct protein-protein contacts at the interface (Fig. 1) (PDB IDs: 1AXM, 2AXM) [45]. Although for obtaining this crystal structure, HE dp10 (dp denotes the degree of polymerization) was used, only HE dp5 (PDB ID: 1AXM) and HE dp6 (PDB ID: 2AXM) were resolved due to the high flexibility of HE ends. Binding of HE induces minimal conformational changes on FGF1, despite the fact that the GAG recognition site has many positively charged residues with flexible side chains, which have been shown to be involved in binding HE analogs with various sulfation patterns [45–47].

GAGs can have different sulfation patterns, known as the ‘sulfation code’ [48], which define their structural properties, molecular recognition and functional activity [49]. Based on available crystallographic data, it has been suggested that FGF1 molecules (FGF1_A_ and FGF_B_, for chain A and B, respectively) selectively recognize the sulfation motif GlcNS-IdoA2S-GlcNS (GIG), while FGF2 selectively recognize IdoA2S-GlcNS-IdoA2S (IGI) [50]. In a recent study by Muñoz-García *et al*., five HE dp6 derivatives (dp6 for hexamers) with different sulfation patterns were obtained, and their influence on FGF1-mediated mitotic activity, and FGF1-HE binding affinities were measured and ranked [51]. The authors also distinguished two continuous sub-sites in the FGF1 binding site, where one sub-site (the large sub-site) is responsible of binding three sugar units constituting the GIG motif. Interestingly, binding affinity and mitotic activity of these derivatives did not correlate [52], which could be related to the fact that the formation of a symmetric FGF-FGFR-HE 2:2:2 complex is responsible for the biological activity, while the FGF1-HE-FGF1 sandwich structure is formed in the absence of the FGFR [32, 51].

The aim of our methodological studies was to use this peculiar FGF1_A_-GAG-FGF1_B_ sandwich complex for which abundant experimental data are available as a model system to drill down on its molecular recognition properties and, thus, to characterize in detail the efficiency and limitations of docking and MD-based approaches. For these purposes, we investigated: *i)* the influence of protein conformational space sampling on docking results; *ii)* the capability of MM-PBSA and free energy perturbation (FEP) approaches to qualitatively and quantitatively distinguish between docking solutions with fine-tuned sulfation pattern differences in comparison to experimental data; *iii)* the predictive power of site-mapping. This technique has been demonstrated in a variety of protein–ligand systems to provide comparable or even better performance in predicting key residues involved in recognition than the “docking top-ranked pose” approach. Agostino *et al*. have recently reported the application of docking and mapping techniques to validate available structural data on protein-GAG systems [53].

From the results we obtained, it can be concluded that protein conformational space analysis is relevant for proper prediction of intermolecular interactions by docking. When compared to experimental values, MM-PBSA free energy calculations were found to have a limited predictive power in scoring the docking solutions obtained by two principally different docking approaches for structurally very similar GAGs. Meanwhile, FEP could qualitatively distinguish between binding energies of GAG derivatives differing in a single sulfate group. The predictive value of docking and MD approaches seems to be enhanced when these techniques are applied in combination with the site-mapping methodology. Despite the above-mentioned challenges for computational treatment of protein-GAG systems and, therefore, for *a priori* prediction of their molecular recognition properties, our results show that the application of several complementary techniques allows to best dissect the challenging nature of GAG-protein interactions. The results obtained here can be useful for the further development and improvement of specific approaches focused on molecular recognition studies of these systems and their rational engineering.

## Materials and methods

### Docking

#### Autodock

The three available structures of FGF1 in complex with HE (PDB IDs: 1AXM, 2AXM, 3UD9) were used for our studies. The crystal structure of FGF1 in complex with HE dp5 (PDB ID 1AXM, 3.0 Å) was used to obtain the unbound protein conformations used for the docking calculations. Prior to docking, the FGF1 receptor protein structures (FGF1_A_/FGF1_B_, chain A and B, respectively) were extracted from the crystallographic complex and optimized by MD (see protocol used below). Representative conformations of FGF1_A_and FGF1_B_ were obtained by clustering (see below) and minimized in MOE [54] using default parameters. These representative structures were used as receptor in the docking calculations. The GAG ligands HE dp5, HE dp5* (in this HE derivative, the second IdoA2S from the reducing end was substituted to IdoA), HE dp6 and its derivatives were modelled in AMBER14 [55] using charges from the GLYCAM06 force field [56] and from the literature for sulfate groups [57]. Docking calculations were performed with Autodock 3 (AD3) [58] with a spacing grid of 0.375 Å. GAGs were treated completely flexible and the protein receptor rigid. The complete protein receptor surface was used for docking. The Lamarckian genetic algorithm with an initial population size of 300 and a termination condition of 10^5^ generations or 9995 × 10^5^ energy evaluations were used. A total of 10^3^ independent runs were performed for each docking experiment.

#### DMD

Dynamic Molecular Docking (DMD) [59], a targeted molecular dynamics (tMD) docking method specifically developed for protein-GAG systems that considers receptor flexibility and explicit solvation, was also used to dock HE and its derivatives to FGF1. The geometrical parametrization needed for DMD (see reference [59] for details) was based on the HE binding region of PDB ID 1AXM. Thus, the Cα atom of FGF1 residue Ile 56 was chosen as *core atom*. Selection of the *focus point* yielded a *tMD target distance* of 30 Å. Each DMD docking experiment involved 100 independent runs.

#### Molecular dynamics

The FGF1-GAG complexes obtained by docking (AD3 and DMD) were optimized by MD simulations carried out in AMBER14 using ff99SB force field parameters for the protein and GLYCAM06 for the GAGs. Each complex was solvated in a TIP3P octahedral periodic box with a minimal distance to the periodic box border of 8 Å, and counterions were used. Two energy-minimization steps were carried out: 0.5 × 10^3^ steepest descent cycles and 10^3^ conjugate gradient cycles with harmonic force restraints on solute atoms, then 3 × 10^3^ steepest descent cycles and 3 × 10^3^ conjugate gradient cycles without constraints. The system was then heated up to 300 K for 10 ps, equilibrated for 50 ps at 300 K, 10^6^ Pa in isothermal isobaric ensemble (NPT) and, finally, a 20 ns productive MD run was carried out in NTP. The SHAKE algorithm, a 2 fs time integration step, an 8 Å cutoff for non-bonded interactions and the Particle Mesh Ewald method were used. GAGs pyranose rings were harmonically restrained for consistency with the experimental data from the analyzed crystal structures. Moreover, although it is known that the used charges [57] can induce instabilities in the equilibrium of IdoA2S ring conformation [60], the time scales of the MD simulations used for post-processing of the docking poses are for practical reasons significantly shorter than the μs time scales required for the establishment of the equilibrium for the pyranose ring interconversion [18]. For the simulation of HE dp6 and its derivatives, we used IdoA2S with no substitution (*e.g.* *i-*propyl group as in [51]) at the reducing end in ^1^C_4_ conformation instead of 4-deoxy-IdoA2S with the distorted ring, considering this approximation relevant for modelling the interactions between long HE chain and FGF1, where the contacts with the protein are established by the internal part of the GAG, which is not deoxidized. Such approximation induced the changes in the glycosidic linkage adjacent to the IdoA2S at the non-reducing end in comparison to the experimental structure with 4-deoxy-IdoA2S.

#### MM-GBSA

The Molecular Mechanics Poisson-Boltzmann Surface Area method with Generalized Born approximation model (MM-GBSA) implemented in AMBER was used for free energy calculations, per residue and residue pairwise decomposition using igb = 2 model for 100 frames evenly distributed in the last nanosecond of the productive MD run.

#### Free energy perturbation

The FEP method included two steps: *i)* electrostatic interactions were gradually turned off; *ii)* van der Waals radii of chargeless atoms were decreased to 0. A sulfate group in the second sugar unit (IdoA2S) was perturbed to hydroxyl group for unbound and protein-bound HE dp5. The free energy difference between these two states was calculated using the thermodynamic integration approach at discrete points of the coupling parameter λ, which was variated from 0 to 1 and then back from 1 to 0 with a 0.1 step along the path. Simulation for each λ value was equilibrated for 200 ps followed by a productive MD sampling for 200 ps. The following parameters were changed from the MD protocol described above: only the first minimization step was employed (*i.e.* 10^3^ using steepest cycles), the heating step was omitted, and a 200 ps equilibration was carried out using a collision frequency of 5 fs.

#### Clustering

The DBSCAN algorithm was used [61]. Representative conformations of each of the receptor protein molecules (FGF1_A_/FGF1_B,_ PDB ID 1AXM) were obtained by clustering of the conformations of FGF1 binding loop residues interacting with HE obtained from the MD simulations. In the case of FGF1_A_, the interacting loop residues were: 17–19, 112–114, 118–122, 126–129, and for FGF1_B_: 18–19, 112–115, 118–122, 127–128. The following parameters were used to cluster the loops: maximal distance of elements in a cluster (ε) and minimal number of neighbours in the cluster (minpoints). Values of (ε = 0.92, minpoints = 2) and (ε = 0.96, minpoints = 2), were used for FGF1_A_ and FGF1_B_, respectively. The 10 most populated clusters of each chain, named “receptor conformations 1 to 10” through the manuscript, were used for docking with AD3. The clustering of the 50 top docking solutions from AD3 and of 100 solutions from DMD was also carried out using the DBSCAN algorithm with ε = 3.0 and minpoints = 4.

#### Site-mapping

The site-mapping was carried out on the data obtained from MM-GBSA free energy decomposition per residue-residue pair and hydrogen bonding (H-bond) obtained from the last nanosecond of MD simulation. MM-GBSA free energy decomposition per residue-residue pair was split into van der Waals (vdW) and total electrostatic (Ele) contributions of interactions between protein and GAG residues. The total electrostatic energy was calculated by summing up polar solvation (GBSA reaction field) and *in vacuo* electrostatic components. H-bonds were calculated by taking all residues in the FGF1-HE complex as possible H-bond donors or acceptors according to AMBER default criteria. In particular, H-bonds were defined by the distance cut-off of 3.0 Å between the heavy atoms of an H-bond donor-acceptor pair and the angle cut-off of 135° between the donor atom, H atom and the acceptor atom. For each H-bond acceptor-donor pair a corresponding time fraction was calculated representing the percentage of frames in which an H-bond was found for that pair. This fraction was used as a parameter for site-mapping. Fractions of the three contributing terms (vdW, Ele, H-bond) were calculated for the structures resulting from the MD simulations of three crystallographic FGF1-HE complexes (PDB IDs 1AXM, 2AXM, 3UD9), for the cluster representatives of the five HE dp6 derivatives in complex with each of the respective receptor conformations (HE1: 13, HE2: 13, HE3: 15, HE4: 11, HE5: 14, where each number indicates the number of independent MD simulations), and for FGF1-HE dp6 derivative complexes predicted by DMD (HE1: 52, HE2: 44, HE3: 54, HE4: 51, HE5: 48) and having at least one sugar residue within 10 Å of the Cα atom of FGF-1 residue Lys 128 to discard the unbound states.

The vdW, Ele and H-bond contributions were individually summed up and normalized for each HE dp6 derivative for the complexes obtained from the AD3 and DMD calculations. Since the crystal structures contained HE of different length (1AXM: dp5, 2AXM: dp6, 3UD9 dp2), the calculated vdw and Ele contributions as well as H-bond fractions were normalized for each sugar ring.

#### Statistical analysis and graphical representation

Data analysis was carried out using in-house scripts and the R package (R Development Core Team 2006).

## Results and discussion

### Free energy analysis of heparin polarity and desulfation

In order to describe the interactions between FGF1 and HE dp5 from the crystal structure of FGF1-HE dp5-FGF1 sandwich [43] (Fig. 1) in terms of free energy, we carried out MD simulations of HE dp5 separately with FGF1_A_ and FGF1_B_. Although the two protein chains bind the same HE dp5 molecule on opposite sides, the HE axis does not act as a true two-fold axis due to the polarity of the sugar chain [44]. The obtained energy values for these two simulated complexes were -68.2 ± 6.7 and -69.7 ± 9.7 kcal/mol suggesting a quantitatively insignificant difference to discriminate HE polarity in terms of the orientation to reducing/nonreducing end. In this analysis, among the 10 FGF1 residues with the most favorable contributions to HE dp5 binding, 8 were found in common for both chains (Supplementary Table 1) meaning that most protein residues responsible for HE dp5 binding are the same independently of its polarity. In the simulation of the sandwich FGF1/HE dp5/FGF1, the free energies corresponding to the binding of HE dp5 by FGF1_A_ and FGF1_B_ are -58.2 ± 9.6 and -60.8 ± 10.2, respectively; whereas for both subunits is −129.7 ± 14.0. The energies obtained for the parts of the FGF1-HE dp5-FGF1 complex in comparison to the FGF1-HE dp5 complex are less favourable, though the differences are statistically insignificant. These differences can be attributed to the fact that in the absence of one of the FGF1 molecules, the HE molecule is not anymore shared between two molecules of FGF1 yielding electrostatic interactions to be better optimized for a separate FGF1-HE dp5 complex.

**Fig. 2:**
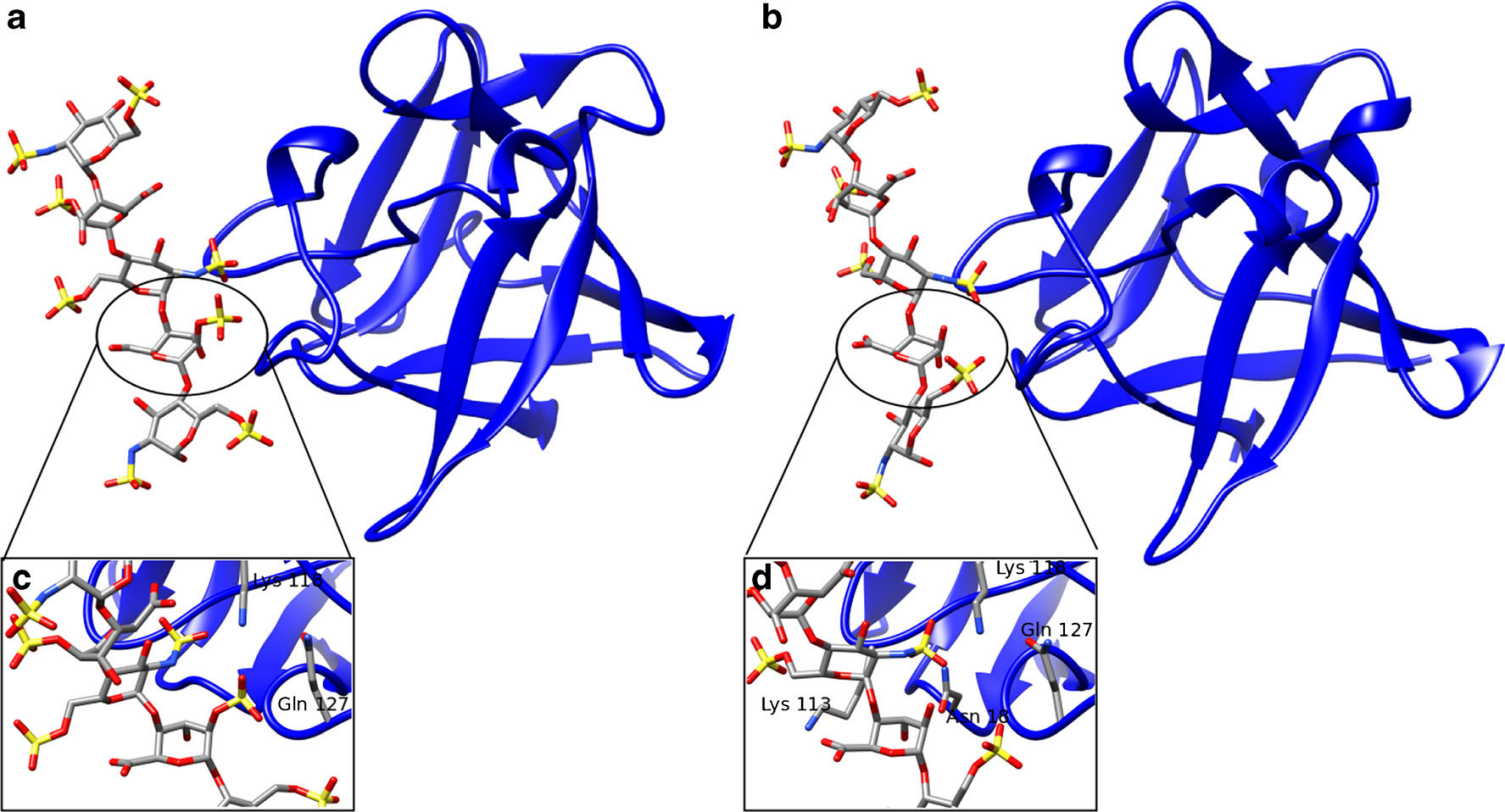
Representative structures of the complexes (**a**) FGF1-HE dp5 and (**b**) FGF1-HE dp5* obtained from the MD simulations. The protein is represented in cartoon and the HE variants in sticks. The protein residues interacting with (**c**) IdoA2S of HE dp5 and (**d**) IdoA of HE dp5* are labelled, and their side chains are shown in grey sticks

Furthermore, we carried out the simulation of FGF1 with the HE dp5 variant from FGF1_A_ in which the second IdoA2S from the reducing end was substituted to IdoA by perturbing the sulfate group into a hydroxyl group (HE dp5*). For the complexes of FGF1 with HE dp5 and with HEdp5*, the crystallographically observed binding mode was retained in the simulation (Supplementary Table 2). In the FGF1-HE dp5 complex, this IdoA2S residue establishes contacts with Lys 118 and Gln 127 through its sulfate group (Fig. 2). In the desulfated variant, IdoA has contacts with Lys 113 through its carboxyl group, and the hydroxyl group establishes contacts with Asn 18. The Lys 118, which is in contact with the sulfate group in IdoA2S in HE dp5, interacts with the central GlcNS6S in the HE dp5*. The desulfation of IdoA2S does not influence the stability of the complex through the MD trajectory in terms of the ligand’s fluctuations in the binding site. The calculated free energy of FGF1-HE dp5* interaction is less favorable (-13.0 ± 10.2 kcal/mol), and the per residue decomposition finds the 9 out of 10 most contributing residues to be the same as with HE dp5 though with different contributions (Table 1). However, as it is to be expected, the contributions of Lys 118 and Gln 127 decreased due to the loss of the sulfate group.

**Table 1:** Residues with the top 10 contributions to ΔG for the complexes FGF1-HE dp5 and FGF1-HE dp5*

Residue	FGF1-HE dp5 ΔG_binding_ (kcal/mol)	Residue	FGF1-HE dp5* ΔG_binding_ (kcal/mol)
Lys 118	-12.7	Lys 112	-8.4
Lys 113	-9.0	Lys 113	-7.5
Lys 128	-7.7	Lys 118	-7.3
Lys 112	-7.2	Arg 122	-7.3
Arg 122	-7.2	Lys 128	-6.0
Gln 127	-6.4	Asn 18	-3.6
Asn 18	-4.6	Gln 127	-3.2
Ala 129	-3.2	Ala 129	-1.4
Arg 35	-1.2	Arg 119	-1.2
Lys 9	-1.1	Arg 35	-1.1

Therefore, MM-GBSA free energy calculations and per residue decomposition are sensitive enough to qualitatively reveal a single desulfation event in the GAG ligand. In order to estimate the quantitative accuracy of our MM-GBSA calculations, we applied FEP. We perturbed the same sulfate group into a hydroxyl group (Supplementary Figures 1a, b, c). First, the charge of the sulfate group was switched off; second, the van der Waals potential was switched off, and a soft-core potential was applied for better convergence of disappearing and appearing atoms; third, an H atom was made to appear, and the potentials were switched on again. The loss of the sulfate group induces van der Waals potential energy decrease, and this cannot be fully compensated by the appearance of the OH group, which is substantially smaller than the sulfate group. The obtained energies from these FEP steps show the significant change of the electrostatic interactions in the system induced by desulfation (Table 2). The contributors to this locally changed electrostatic characteristic of the system are *(i)* the direct effect of the changes in charge-charge interactions between protein and sugar groups, and *(ii)* the dramatic rearrangements in the solvation shell near the substituted group (Supplementary Figure 2).

**Table 2:** ΔG differences and their standard deviations obtained at each FEP step as well as in total

FEP Step	ΔG (kcal/mol)
ΔG Step 1	0.8 ± 10
ΔG Step 2	2.3 ± 17
ΔG Step 3	0.5 ± 6
ΔG Total	3.6 ± 21

Both MM-GBSA and FEP are able to unambiguously distinguish a stronger binding for the fully sulfated HE dp5 in comparison to HE dp5*. The overestimation of the obtained difference by MM-GBSA in comparison to more accurate FEP might be attributed to the neglect of explicit solvation effects in the implicit solvent model used in MM-GBSA.

### Docking HE dp5 to the ensemble of FGF1 structures and its sensitivity to desulfation

The next question we aimed to address was how taking into account the receptor conformational space can influence HE dp5 docking in terms of localization of the ligand on the surface of the receptor (placement) and in terms of the calculated free energy of interaction between FGF1 and HE dp5 for the obtained solutions (scoring). First, we obtained an ensemble of FGF1 structures from the MD simulation: a total of 11 and 10 clusters were obtained for FGF1_A_ and FGF1_B_, respectively. Then we clustered them based on the RMSD values of the HE binding loop (see Materials and Methods) and extracted representative structures from the 10 most populated clusters. We used these FGF1 representative structures for docking with HE dp5 and HE dp5* as ligands and obtained 13 and 18 clusters, respectively. Out of these docking solutions, there were one and three clusters not located in the experimentally determined binding region for HE dp5 and HE dp5*, respectively. The representative docking solutions for each cluster were further analyzed by an MD simulation followed by MM-GBSA free energy calculations. Interestingly, for the HE dp5 cluster located far from the experimentally determined binding region (receptor conformation 1, cluster 2, see Table 3) corresponded to the most unfavorable energy of interaction among all analyzed clusters (Supplementary Figure 3). In general, AD3 was able to predict the HE binding site, which could also be clearly distinguished by simple electrostatic potential calculations [57]; however, the choice of a proper receptor conformation prior to docking could be crucial for better performance. It is also noteworthy that, with the exception of receptor conformation 1, clustering only identified a single cluster for all other receptor conformations. As expected, all these docking solutions were found near the binding site; however, some were additionally extended in space (Supplementary Figure 4), meaning that not all HE dp5 sugar units are required for establishing contacts with the protein binding site. The same elongation could be observed for HE dp5* for individual clusters even more evidently (Supplementary Figure 5).

**Table 3:** ΔG values for HE dp5 and HE dp5* obtained from the MD simulations of AD3 docking solutions

FGF1 receptor conformation	FGF1-HE dp5 ΔG (kcal/mol)	^1^ RMSatd (Å) of ligand with respect to X-ray	Ligand orientation with respect to X-ray chainA	FGF1-HE dp5* ΔG (kcal/mol)	^1^ RMSatd (Å) of ligand with respect to X-ray	Ligand orientation with respect to X-ray chainA
1	-67.1	6.0	perpendicular	-28.9	6.9	parallel
	-26.5	26.5	n.a.	-52.2	4.4	parallel
	-59.3	16.7	antiparallel	n.a.		n.a.
2	-81.5	5.1	parallel	-81.9	6.0	parallel
	-89.2	5.9	parallel	-43.9	28.6	---
3	**-**76.5	4.7	perpendicular	-74.9	5.0	parallel
4	-74.7	7.2	perpendicular	-22.9	7.2	antiparallel
	n.a.		n.a.	-75.4	5.2	parallel
5	-103.1	5.5	parallel	-82.3	5.2	parallel
6	-59.9	5.6	parallel	-26.5	5.9	perpendicular
	n.a.		n.a.	-69.2	4.7	parallel
7	-59.4	4.2	parallel	-72.6	5.1	parallel
8	-77.9	5.0	antiparallel	-46.7	3.2	parallel
	n.a.		n.a.	-58.7	7.2	parallel
	n.a.		n.a.	-71.5	5.0	parallel
	n.a.		n.a.	-37.1	6.4	parallel
9	-56.7	5.4	perpendicular	-45.7	4.9	parallel
	n.a.		n.a.	-54.7	4.3	parallel
10	-89.2	4.4	perpendicular	-41.7	4.5	antiparallel

Ligand orientation was determined relative to the axis of HE dp5 in its crystallographic complex with FGF1_A_ (PDB ID 1AXM)

^1^ Root mean square atom type distance (RMSatd) is used in the same way as regular RMSD, with the  exception that it pairs up spatially close atoms of the same type.

**Fig. 3:**
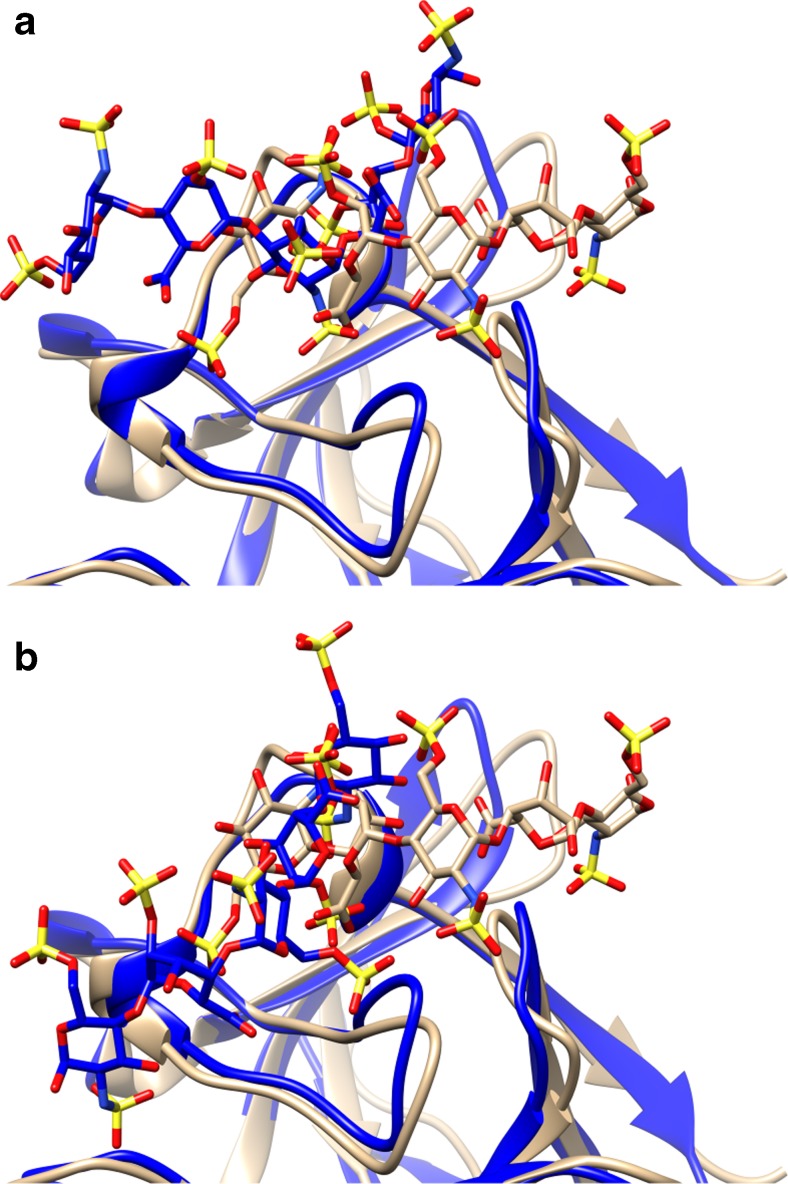
Comparison of the best-scored docking solution for HE dp5 (with receptor conformation 5) with the crystal structure: (**a**) FGF_A_ and (**b**) FGF_B_. Protein is shown in cartoon and ligand in sticks. *Blue* is used for the crystal structure and *beige* for the docking solution

From all obtained docking solutions, the one corresponding to the receptor conformation 5 was found to have significantly more favorable energy in comparison to the other docking solutions and, noticeably, to the free energy value obtained for the crystal structure (Table 3). In the structure corresponding to this docking solution, HE is shifted by one disaccharide unit and has the same polarity as the HE in the crystal structure (Fig. 3a), while the interacting protein residues differ only by 0.9 Å heavy atoms RMSD in comparison to FGF1_A_ of the crystal structure. When compared to FGF1_B_ of the crystal structure, the RMSD obtained for the protein residues is 1.0 Å, and the HE dp5 has opposite polarity. Though in this case a shift in the position of the docking solution is also observed, the GIG motif occupies almost the same area as in the crystal structure (Fig. 3b). This is consistent with the hypothesis that the FGF1 binding site can be divided into two sub-sites. The crystal structure, reflecting a dimer, implies a conformation of HE dp5 where the GIG binding motifs bind to the sub-site suited for the three sugar units GIG motif on both monomers. In our AD3 results, the highest affinity docking solutions cover both sub-sites when only one monomer is taken into account.

For HE dp5*, all docking solutions are clustered within or near the HE binding site. There are significantly more clusters obtained than for HE dp5, which might indirectly suggest the importance of the missing sulfated group in HE dp5* for the binding specificity to FGF1. Free energy calculations for HE dp5* solutions yield on average ~ 15 kcal/mol less favorable energy than for HE dp5, which can be attributed to the net electrostatics change due to the desulfation. This is consistent with the 13 kcal/mol value obtained for the desulfation carried out directly in the crystal structure. Interestingly, the best scored solution for HE dp5* also corresponded to the receptor conformation 5, and its energy was more favourable than for HE dp5 in the crystal structure. The structure polarity remained in this case the same as in the crystal structure. In comparison with receptor conformation 5 in complex with HE dp5, both HE chains overlap very similarly in the FGF1 binding site though with opposite polarity (Supplementary Figure 6). This can be due to the fact that HE dp5* contains only one fully sulfated GIG motif, and this motif is responsible for making specific contacts with the large sub-binding site formed by residues 113–122 and additionally Asn 18.

We structurally analyzed those docking results obtained with the different FGF1 conformations presenting the most favorable binding energies for both HE dp5 and HE dp5* (receptor conformations 2, 3, 4, 8). In the case of receptor conformation 2, HE dp5* is localized similarly in the FGF1 binding site presenting the sulfated GIG to the large sub-site. HE dp5 binding pose corresponding to the most favorable binding energy is shifted in comparison to HE dp5* and displays opposite polarity. HE dp5 and HE dp5* docking solutions have comparable binding affinity when obtained in complex with receptor conformations 3 and 4. In the complexes with receptor conformation 3, both HE dp5 and HE dp5* docking solutions adopt the same polarity. HE dp5* occupies a very similar position to the solutions with receptor conformations 2 and 5, and it binds to the larger sub-binding site with the sulfated GIG motif. The complexes with receptor conformation 4 also show the same polarity for both HE ligands. However, while HE dp5* binds the larger sub-site with its non-reducing end containing GIG, HE dp5 binds the same site with its reducing end GIG motif. Both HE dp5 and HE dp5* have the remaining two sugar units extended away from the binding site. Finally, the complexes with receptor conformation 8 also show high affinity for HE dp5 and HE dp5*. The binding poses for the two HE variants show similarity to those found for receptor conformation 4: HE dp5* is bound in almost the same pose with the same polarity as in receptor conformation 4 complex, and HE dp5 is bound with opposite polarity, extending its non-reducing end to the binding site.

In summary, the results show that the MM-GBSA approach applied to AD3 docking solutions obtained with the consideration of receptor flexibility are noisy, lack significant relation between the RMSD to the experimental structure and the corresponding score, and represent a serious challenge for clear interpretation. Highly similar binding poses are very challenging to score and rank properly, and binding affinity differences for HE dp5 and HE dp5* calculated with MM-GBSA are within the margin of error. Nevertheless, all the predicted high affinity docking poses imply the importance of the GIG motif for establishing the interactions in the binding site and, therefore, for FGF1-HE complex formation suggested by experiments [50].

### Docking HE dp6 variants with different sulfation patters to FGF1

**Fig. 4:**
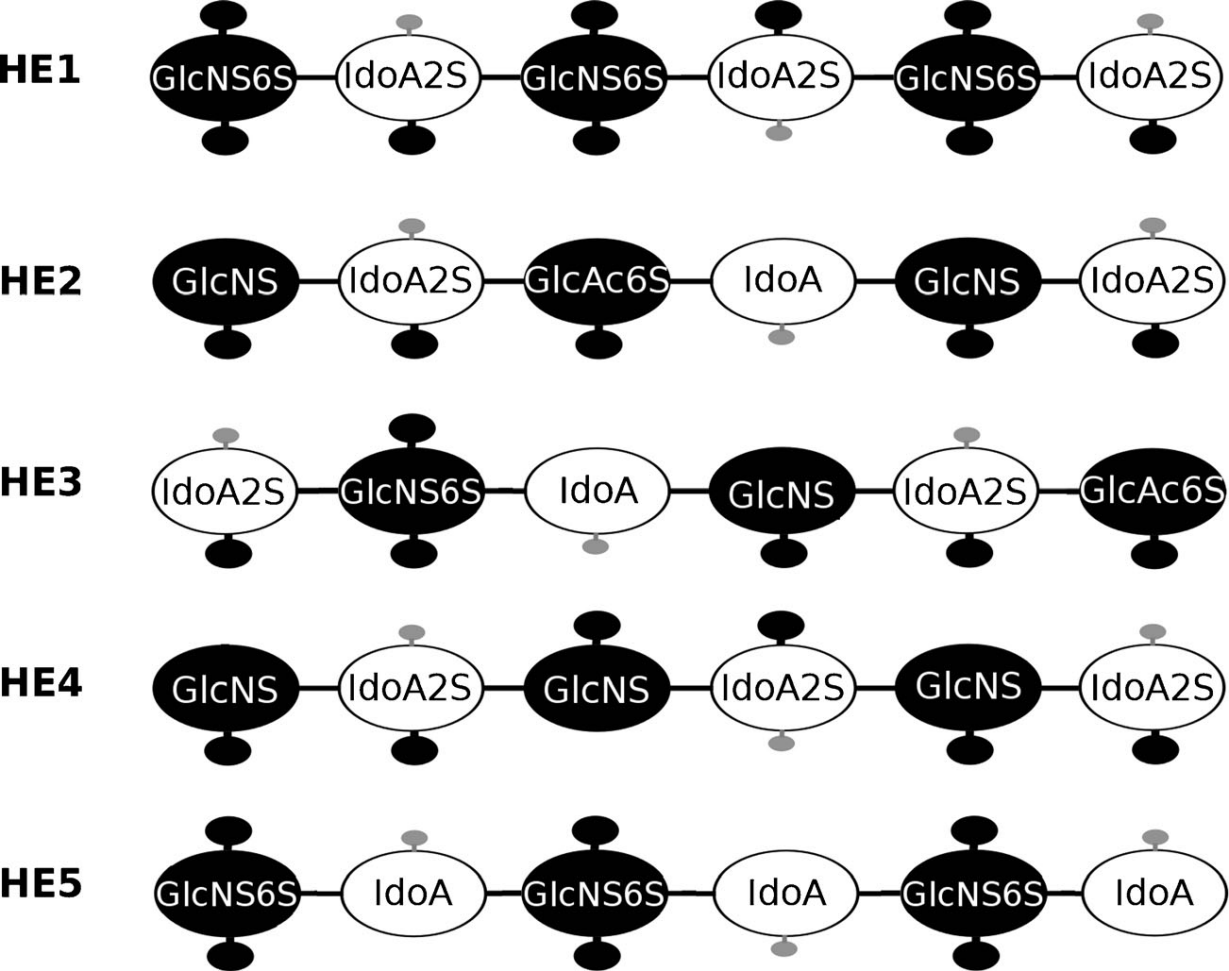
Schematic representation of five HE dp6 derivatives with different sulfation patterns obtained by Muñoz-García *et al* [39]. Black and white big circles represent GlcNS and IdoA, respectively. Small black and gray circles represent sulfate and carboxyl groups, respectively. All HE derivatives are shown from non-reducing to reducing end from left to right

We applied the same docking and scoring procedure to five HE dp6 derivatives (Fig. 4) for which there are available experimental data on FGF1-GAG binding affinity ranked as following: HE1 > HE2, HE4 > > HE3 > > HE5 [50]. We ranked the MM-GBSA free energies obtained from the MD simulation of the AD3 docking solutions for the HE dp6 variants (Supplementary Table 3), and we recorded the times that each ligand scored in each of the five ranking positions from 1 to 5 (Supplementary Table 4). When the docking solutions were clustered into more than one cluster, the most favourable ΔG value was used for ranking. The AD3-based ranking obtained was HE1 > HE3 > HE2 > HE5 > HE4, which shows that this approach managed to properly identify HE1 as the strongest binder but failed to rank the other ligands.

The same procedure was carried out for docking solutions obtained by DMD (see Materials and methods for details), where the flexibility of the receptor is already implicit. Therefore, 100 solutions for each HE dp6 variant with the same initial receptor conformation were considered for analysis. When only the most favourable energy values were taken into account, no agreement with the experimental ranking was achieved. However, when the whole set of the DMD solutions was considered, the experimental ranking was reproduced: HE1 > HE4 > HE2 > HE3 > HE5 with the statistical significant differences according to the *t*-test (p-value < 0.05) obtained for the pairs: (HE1, HE5), (HE1, HE3), (HE2, HE5), (HE4, HE5). These results should be interpreted carefully due to the fact that 100 solutions could be still too few to quantitatively distinguish all the calculated binding free energies distributions (Supplementary Figure 7), though this number of DMD solutions was optimized in our previous work to achieve convergence in several test systems similar to FGF1-HE dp6 in terms of number of contacts between ligand and receptor [59]. The findings obtained with the DMD approach suggest that, in order to reliably compare docking results for protein-GAG systems scored by the MM-GBSA-based approach, one should take into account an ensemble of ~10^2^ solutions, whereas a classical approach considering only the best scored poses is rather expected to fail for such systems as we see in the case of AD3, because it deals with arbitrarily chosen representatives from quite broad distributions. These findings support previous work by us [13] and others [14] suggesting that protein-GAG interactions should not be generaly related to just a single predominant binding pose but could be rather considered and understood in terms of multipose ensembles. Therefore, methods such as the site-mapping approach [53] could be quite beneficial for the analysis of protein-GAG systems.

### Site-mapping of FGF1 residues participating in the recognition of HE dp6 derivatives

**Fig. 5:**
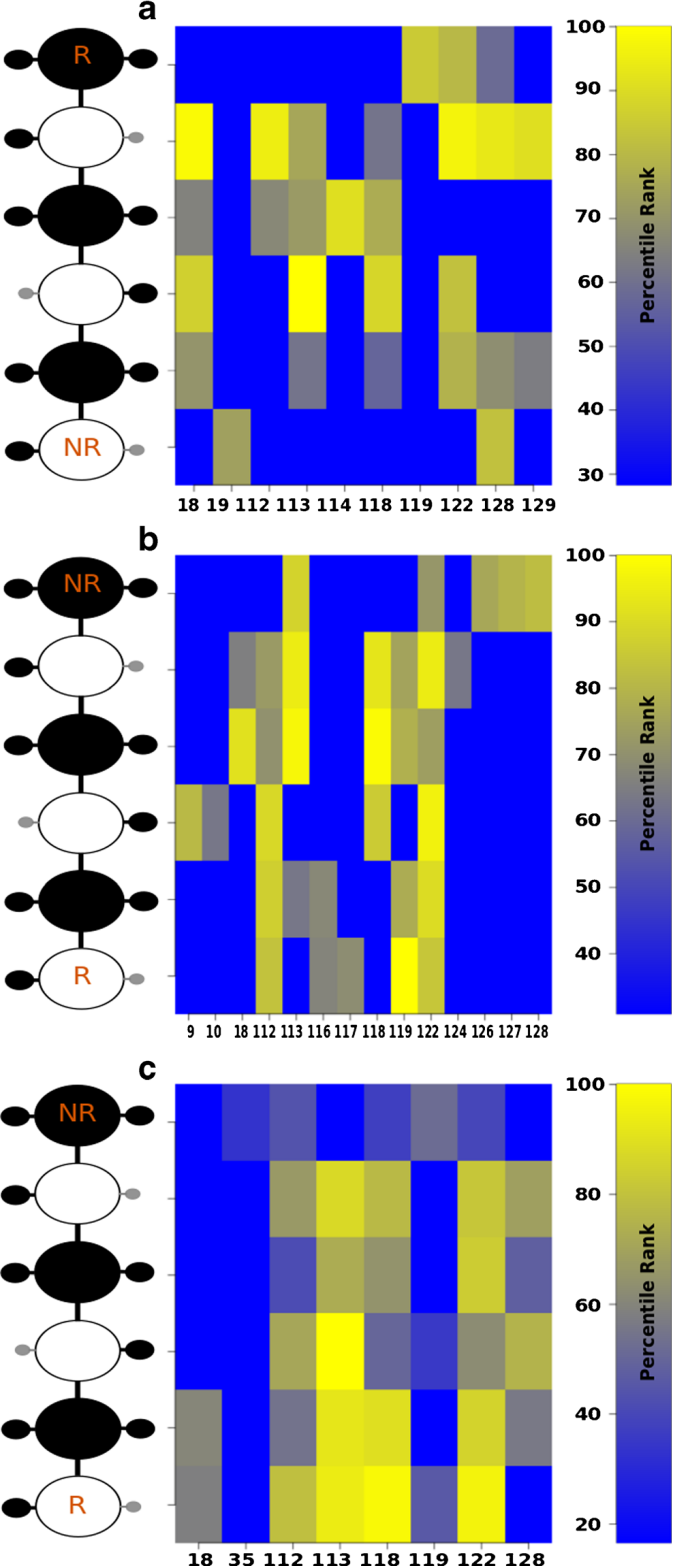
H-bonding pattern observed for HE1 dp6 in the (**a**) the crystal structures, (**b**) AD3 complexes and (**c**) DMD complexes. Protein residue numbers and GAG residues are indicated in x- and y-axis, respectively. NR and R indicate the GAG non-reducing and reducing ends, respectively

To further analyze the structural basis for binding affinity and specificity differences of the six HE dp6 derivatives, we used site-mapping and determined the most relevant residues for binding and recognition by FGF1 in terms of several contributions to the free energy of interactions: hydrogen bonding (H-bonds), electrostatics (Ele) and van der Waals (vdW). We first constructed site-maps using crystallographic data obtained from the PDB for three structures of FGF1 with HE dp2, dp5 and dp6 (see Materials and methods). Based on these structural data, we identified a total of 33 H-bonds between HE and FGF1 established by the following 10 residues: Asn 18, Gly, 19, Lys 112, Lys 113, Asn 114, Lys 118, Arg 119, Arg 122, Lys 128, Ala 129. According to the calculated H-bonding frequencies (Fig. 5a), Asn 18, Lys 112, Lys 113, Arg 122 and Arg 128 form the most frequently observed H-bonds with IdoA2S units of HE dp6, highlighting the particular importance of IdoA2S sulfate groups in binding, which is in good agreement with previous SPR measurements [50]. Similarly, from the MD simulations of these three FGF1-HE crystal structures, only the central GlcNS was comparable to IdoA2S units in terms of H-bonding frequencies. When analyzing the H-bonding frequencies obtained in the docking simulations of HE1 dp6, we found a higher number of H-bonds than for the experimental structures, which is to be expected as AD3 yields various docking poses. Therefore, we chose the 33 most frequent H-bonds for the further comparison (Fig. 5b). These bonds were established by 14 residues (Lys 9, 10, Asn 18, Lys 112, Lys 113, Ser 116, Cys 117, Lys 118, Arg 119, 122, His 124, Gly 126, Lys 128), which included 7 residues identified in the top 10 residues obtained from the simulations of the experimental structures. The most frequently observed H-bonds established by Asn 18, Lys 113, Arg 119, Arg 122 were more equally distributed between GlcNS and IdoA2S sugar units than in the case of crystal structures. Similarly, the analysis carried out for DMD shows that 8 residues established the 33 most frequent H-bonds: Asn 18, Arg 35, Lys 112, Lys 113, Lys 118, Arg 119, Arg 122, Lys 128, containing 7 which match to the top 10 obtained from the simulations of the experimental structures. Lys 113, Lys 118 and Arg 122 clearly stand out in both DMD results and in the crystal structures (Fig. 5c).

**Fig. 6:**
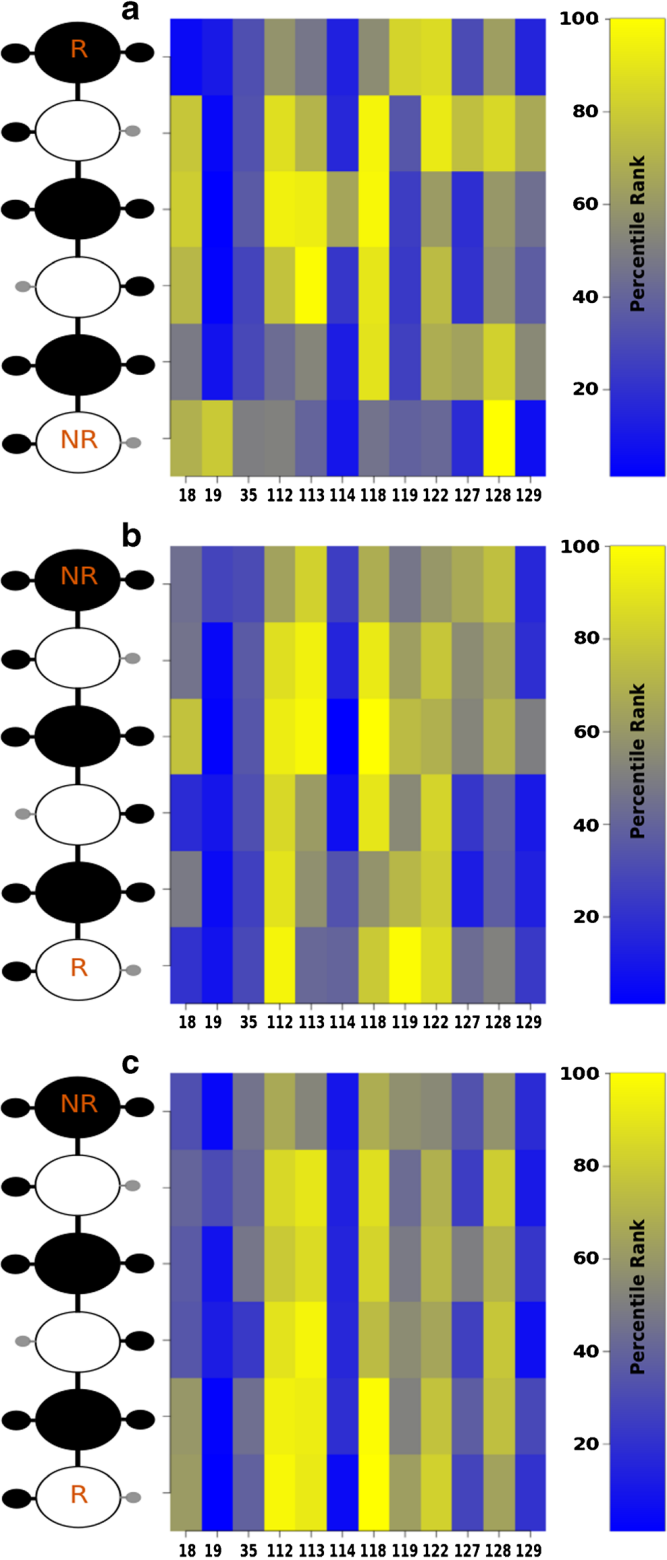
Electrostatic interactions observed for HE1 dp6 in the (**a**) the crystal structures, (**b**) AD3 complexes and (**c**) DMD complexes. Protein residue numbers and GAG residues are indicated in x- and y-axis, respectively. NR and R indicate the GAG non-reducing and reducing ends, respectively

In the analysis of the electrostatic interactions (Ele) between the protein residues and sugar units of the crystal structures and HE dp6 derivatives, the data obtained were very similar to the one from H-bonding analysis with the highest contribution corresponding to the residues: Asn 18, Lys 112, Lys 113, Lys 118, Arg 122 and Lys 128 (Fig. 6a). The data obtained by AD3 and DMD with HE1 suggest the highest Ele contributions from Lys 112, Lys 113, Lys 118, Arg 119 and Arg 122 for the first (Fig. 6b), and Lys 112, Lys 113, Lys 118, Arg 122, Lys 128 for the latter (Fig. 6c). Therefore, both docking approaches were able to map well the residues with the highest electrostatic contributions.

**Fig. 7:**
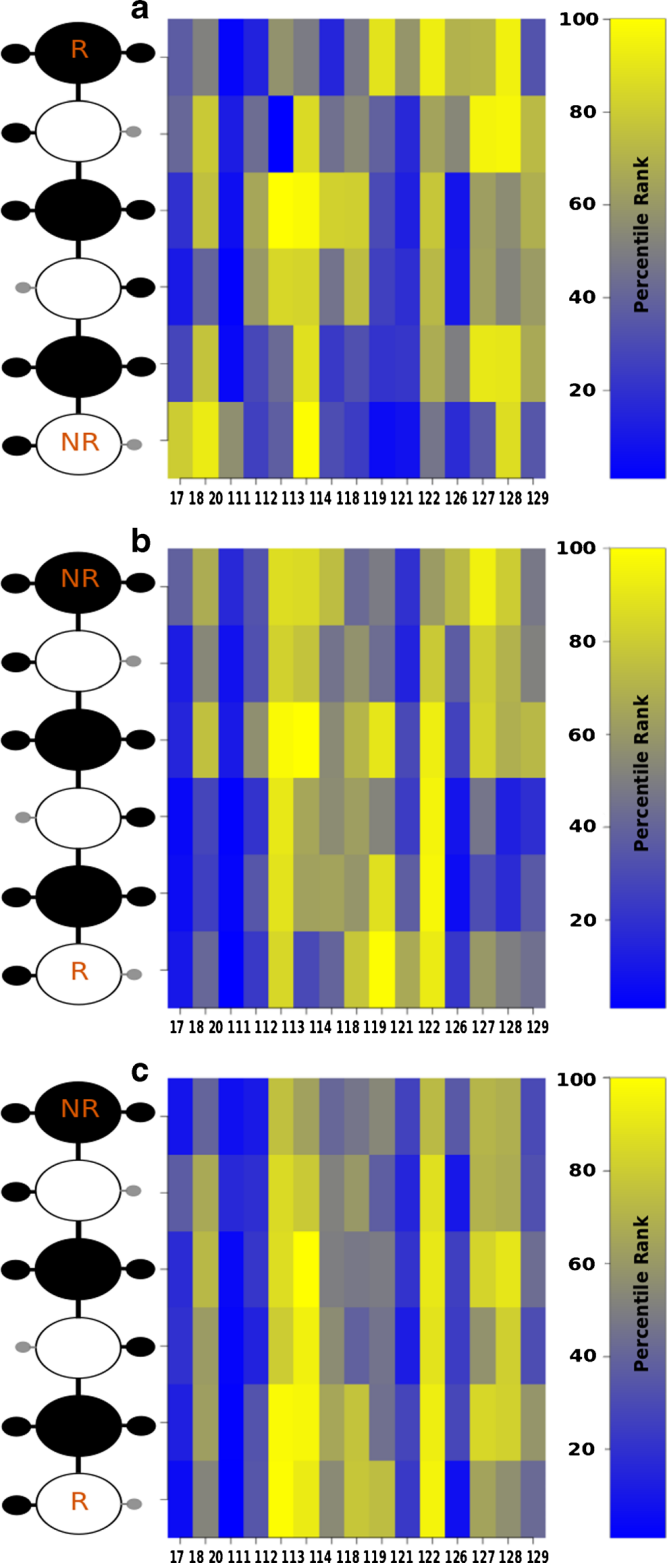
VdW interactions observed for HE1 dp6 in the (**a**) the crystal structures, (**b**) AD3 complexes and (**c**) DMD complexes. Protein residue numbers and GAG residues are indicated in x- and y-axis, respectively. NR and R indicate the GAG non-reducing and reducing ends, respectively

Finally, the crystal structures and HE1 poses obtained by docking were compared in terms of van der Waals (vdW) contributions. From the crystal structures, residues Asn 18, Lys 113, Arg 122, Gln 127 and Lys 128 were identified to be particularly important (Fig. 7a). AD3 yielded highest contributions of Lys 112, Lys 113, Arg 119, Arg 122 and less pronounced contributions for Gln 127 and Lys 128 (Fig. 7b). DMD results suggested rather weak vdW interactions mediated by Asn 18, Lys 112 and Lys 113 and high contributions of Arg 122, Gln 127 and Lys 128 (Fig. 7c). In both docking and crystal structures analysis, most of the vdW contacts are observed to be established with GlcNS sugar units, which are bulkier than IdoA2S.

In general, the site-mapping approach applied to the docking results was successful in identifying the key protein residues binding HE. The correlation analysis between the corresponding datasets of residue pairwise interactions obtained from the crystal structures and the AD3 and DMD docking procedures shows that AD3 performs better than DMD in H-bond prediction. For Ele, the two docking procedures perform equally well, while for vdW DMD performs better than AD3 (Supplementary Table 5).

As a next step, we compared the H-bonds, Ele and vdW site mapping results obtained for the HE1 dp6 and the other HE dp6 derivatives. Although the correlation between the AD3 results obtained for HE1 and HE2 in terms of H-bonds was slightly higher than for DMD, the latter were more informative (Supplementary Figure 8). In particular, according to DMD, the analysis of H-bonds established by HE2 clearly reflects the loss of a sulfate group at the 4^th^ IdoA from the nonreducing end and the strongest contacts made by the remaining two IdoA2S sugar units. The residues involved in H-bonding of the second IdoA2S at the nonreducing end are Lys 112 and Lys 113, while for the IdoA2S at the reducing end residues Lys 118, Arg 119 and Arg 122 take part. The analysis of Ele interactions identifies the same differences in the interacting partners. The analysis of vdW interactions, however, shows that the sulfated GIG motif located at the nonreducing end of HE2 has more abundant and stronger interactions than HE1 (Supplementary Figure 9). At the same time, a weaker vdW interaction can be observed for the fourth IdoA, which results from the loss of a sulfate group. For HE3, DMD is able to identify the loss of sulfation on the third IdoA from the nonreducing end in terms of H-bond interactions (Supplementary Figure 10), while AD3 shows that the GIG motif found at the reducing end is more likely to establish H-bonds as well as vdW interactions (Supplementary Figure 11). Besides the preference for the GIG binding motif, these results indicate that polarity is an important factor underlying FGF1-HE3 dp6 binding, which is in agreement with previous findings [50].

The comparison of HE1 and HE4 in terms of H-bonds and Ele shows a loss of interactions in the case of the 3^rd^ GlcN from the nonreducing end as well as for the fourth IdoA2S with residues Arg 119 and 122 (Supplementary Figure 12). The vdW interactions analysis shows that the GIG motif starting at the nonreducing end interacts weaker with Asn 18, Lys 112 and Lys 113, while the subsequent IGI motif retains its interactions with Lys 112 and Lys 113, and it establishes stronger contacts with Asn 18, Asn 127 and Lys 128 when compared to HE1 (Supplementary Figure 13).

Both AD3 and DMD site mapping results show that HE5 dp6 establishes most of its H-bonds by its GlcNS as could be expected since there are no sulfates on IdoA sugar units (Supplementary Figure 14). Similarly, most Ele and vdW interactions are mediated by GlcNS units.

To check how the site-mapping approach could potentially discriminate between binders and non-binders, we conducted blind docking with AD3 for desulfated HE dp6 (HE-dp6**), which does not contain the important binding GIG motif. Then, we carried out site-mapping analysis for the cluster of solutions obtained in the HE experimental binding site. The correlation data for H-bonds, Ele and vdW interactions (Supplementary Table 5) show that, for all three types of interactions, the lowest correlation is obtained for HE-dp6** with the crystal structure (HE dp6), while the highest correlation is with HE5, which does not show any significant binding to FGF1 [51]. According to the H-bonding pattern (Supplementary Figure 16 a), residues Lys 118, Arg 119 and Arg 122 are the most important for binding HE-dp6**. H-bond formation is more likely with IdoA carboxyl group as opposed to GlcNS in case of HE dp6. Binding pose analysis shows that HE-dp6** is rather partially bound to the FGF1 binding site by two or three monomeric saccharide units either at its reducing or non-reducing end. Interestingly, Ele and vdW interactions analysis (Supplementary Figure 16 b and c) shows that binding occurs at the two ends of the binding site, one localized around Asn 18 and Gly 19, and the other around Lys 128 and Arg 129. The lack of H-bonds in the region of Asn 18 and Gly 19 indicates that there is no specific binding at this region. There is also no significant difference between IdoA and GlcN in terms of vdW interactions, whereas Ele site maps show that the GlcN at the non-reducing end establishes stronger interactions due to the formation of H-bonds. This analysis applied to a non-binder HE-dp6** supports the potential of the site-mapping approach to distinguish binders from non-binders.

Based on our analyses, we conclude that the site-mapping approach represents a valuable method to identify key residues pairs involved in FGF1-HE recognition, as shown for HE1 dp6. Furthermore, this procedure also performs well in distinguishing among HE dp6 derivatives in terms of pairwise interactions. By incorporating MM-GBSA-derived energies from Ele and vdW interactions as well as H-bonds into the site-mapping approach, it is possible to track the effect on binding caused by specific changes in the GAG sulfation pattern. This, however, does not necessarily imply that the approach is able to detect all fine differences in ligand sulfation pattern to unambiguously distinguish their binding modes. The site maps generated by AD3 and DMD are comparable when considering the correlation with the data obtained from the crystal structures in case of HE1 dp6. However, DMD proved to be a more reliable method for identification of the differences in binding distinct HE dp6 derivatives. This might be due to the fact that FGF1-HE recognition is primarily governed by electrostatic interactions predominantly mediated by the long and flexible positively charged side chains of residues at the binding site, which can be very sensitive to the alterations in the ligand structure. Despite its more expensive computational cost, DMD addresses the receptor flexibility issue, whereas AD3 is limited in this regard. At the same time, the drawback for DMD resides on the fact that the information about a putative binding site is required, while AD3 is applicable to blind docking.

## Conclusions

In this work, we apply molecular docking and MD-based approaches to drill down on the molecular recognition particularities of the FGF1-HE system and to evaluate the applicability and sensitivity of the applied methodology. First, we confirm that a thorough analysis of the receptor’s conformational space may be crucial for docking results even for such a small system. We observe that the MM-GBSA approach is not sufficient to distinguish binding polarity of HE dp5 on FGF1, as seen by the insignificant energy differences obtained for each of the protein chains in the FGF1-HE-FGF1 sandwich. However, a single desulfation in HE dp5 leads to a preferred polarity of the bound HE molecule and clear identification of a significant unfavourable change of the calculated binding energy. Quantitatively, this energy change is essentially overestimated by the MM-GBSA approach in comparison to the values obtained by FEP suggesting a crucial role of explicit hydration in such calculations. Molecular docking for both HE dp5 and HE dp5* with a single desulfated group yields noisy results in terms of scoring and ranking when the MM-GBSA approach is applied, which makes a detailed analysis challenging. When comparing the binding ranking obtained for a series of HE dp6 derivatives with available experimental data, AD3 fails to reproduce the experimental ranking order with the exception of being able to identify the best binder, whereas DMD yields a proper though not always significant binding ranking. The combination of the site-mapping approach with both AD3 and DMD appears quite promising for reproducing the experimentally observed interactions in the FGF1-HE system, and also to be able to distinguish the residue pairwise interaction patterns for distinct HE dp6 derivatives as well as potential binders from non-binders. All in all, our model study adds to our understanding of the applicability of available theoretical approaches to the investigation of molecular recognition in protein-GAG systems.

## Electronic supplementary material

Below is the link to the electronic supplementary material.

ESM 1 (PDF 1976 kb)
