# Supplementary material for: Computational drill down on FGF1-heparin interactions through methodological evaluation
Source: Glycoconj J. 2016 Nov 17;34(3):427–40. doi: 10.1007/s10719-016-9745-4 (PMC5487771; doi:10.1007/s10719-016-9745-4)
Supplement: Supplementary file 1 — (PDF 1976 kb) [file 10719_2016_9745_MOESM1_ESM.pdf]

Computational drill-down on FGF1-heparin interactions through methodological evaluation

Sándor Babik, Sergey A. Samsonov, M. Teresa Pisabarro

Structural Bioinformatics, BIOTEC TU Dresden, Dresden, 01307 Germany.

*Corresponding author: MTP. Tel.: +49 (0) 351 463 400 71; Fax: +49 351 463 40087

E-mail: mayte@biotec.tu-dresden.de

**$FGF1_A$ – HE dp5**		**$FGF1_B$ – HE dp5**	
**Residue**	**ΔG (kcal/mol)**	**Residue**	**ΔG (kcal/mol)**
Lys 118	-12.7	Lys 112	-13.1
Lys 113	-9.0	Lys 113	-12.5
Lys 128	-7.7	Lys 118	-7.0
Lys 112	-7.2	Lys 128	-6.8
Arg 122	-7.2	Arg 119	-6.1
Gln 127	-6.4	Arg 122	-5.9
Asn 18	-4.6	Gln 127	-4.9
Ala 129	-3.2	Asn 18	-3.5
Arg 35	-1.2	Ala 129	-3.0
Lys 9	-1.1	Asn 114	-1.5

Supplementary Table 1. Top ten residues with the highest contribution to ΔG in the complexes $FGF1_A$ – HE dp5 and $FGF1_B$ – HE dp5.

**$FGF1_A$ – HE dp5**		**$FGF1_A$ – HE dp5***	
**Residues**	**RMSD, Å**	**Residues**	**RMSD, Å**
Whole HE dp5	2.2 ± 0.5	Whole HE dp5*	2.0 ± 0.4
GlcNS-1	1.4 ± 0.4	GlcNS-1	1.3 ± 0.2
IdoA2S-2	0.7 ± 0.3	IdoAU-2	0.4 ± 0.1
GlcNS-3	1.0 ± 0.2	GlcNS-3	1.0 ± 0.2
IdoA2S-4	0.7 ± 0.3	IdoA2S-4	0.8 ± 0.3
GlcNS-5	1.3 ± 0.3	GlcNS-5	1.3 ± 0.3

Supplementary Table 2. RMSD (all atoms) of the whole HEdp5 and HEdp5* and of their monomeric units in the MD simulation with the reference to the starting experimental structures.

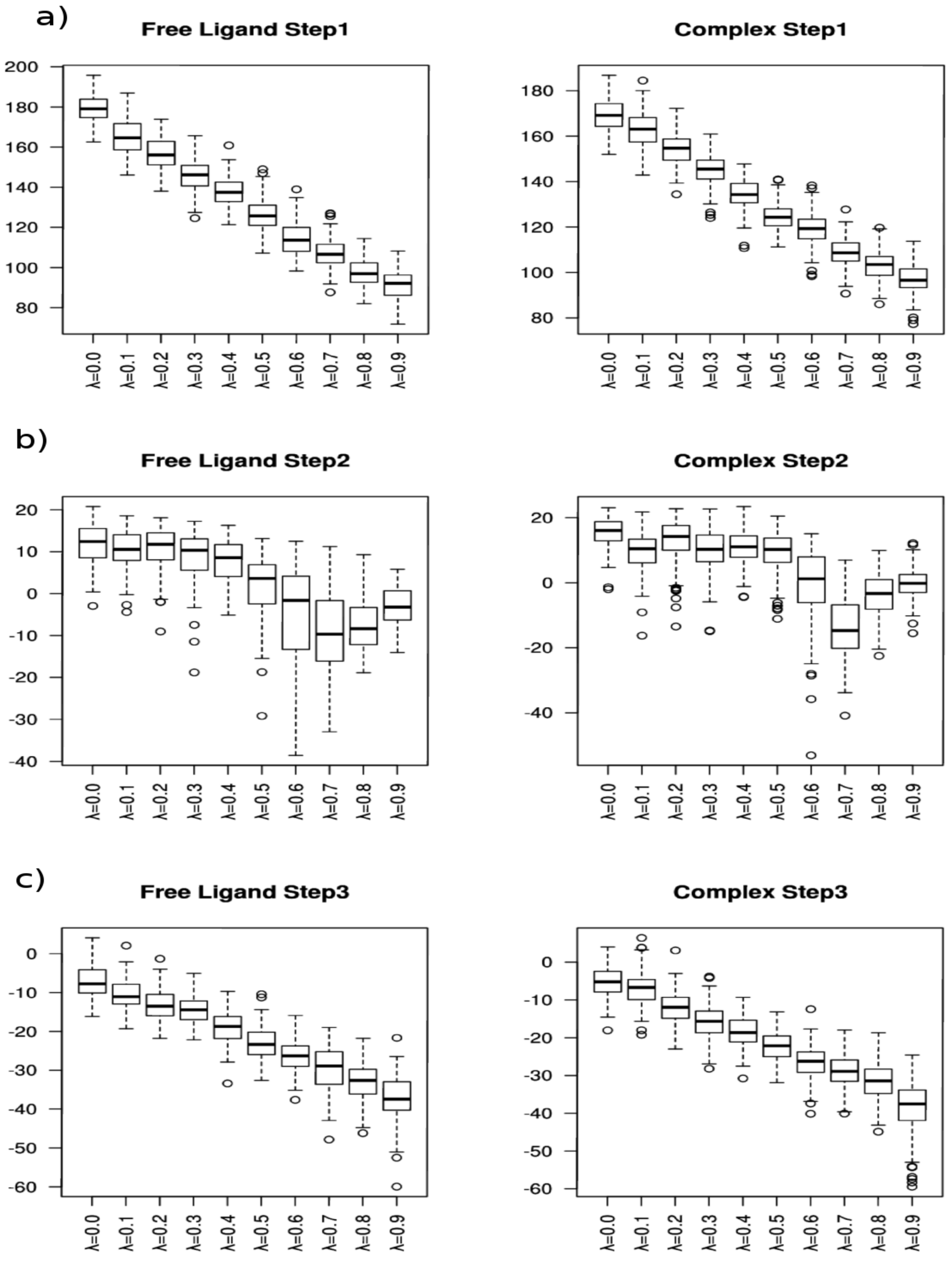

Supplementary Figure 1. In step 1 of FEP (a), the electrostatic interactions are turned off, in step 2 (b) the van der Waals potential is replaced by a soft-core potential allowing for the annihilation of the sulfate group and the appearance of the hydroxyl group, in step 3 (c) the electrostatic interactions are switched back on. Energy gradients (kcal/mol/λ) are shown on the y axis, while the adiabatic lambda (λ) coupling parameter is represented on the x axis.

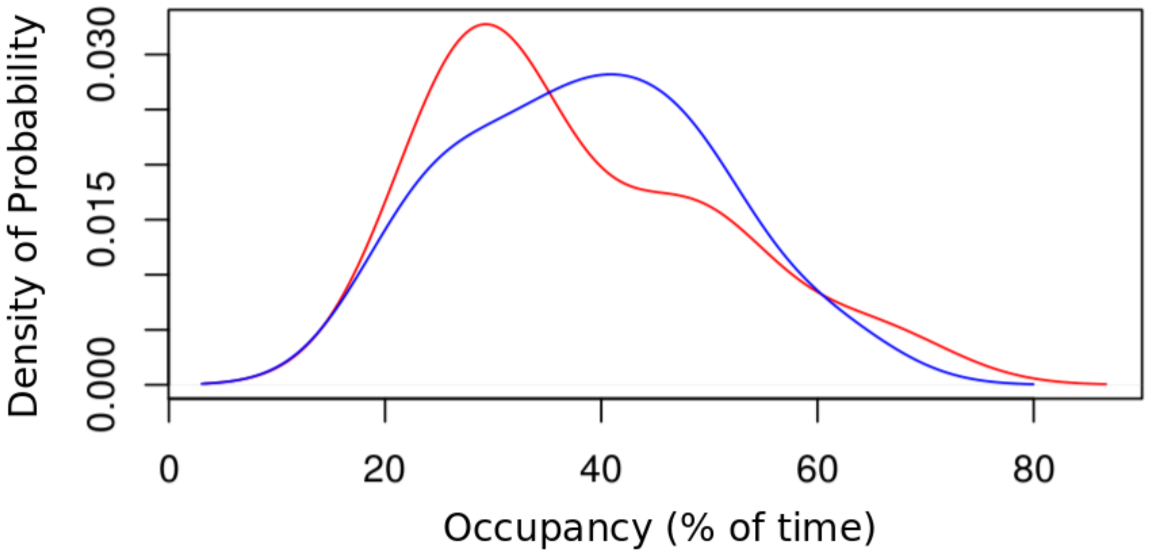

Supplementary Figure 2. Differences in the water shell surrounding IdoA2S (blue) and IdoA (red) calculated in terms of the occupancy of H-bonds formed between the sulftate/hydropxyl group and surrounding water molecules, respectively.

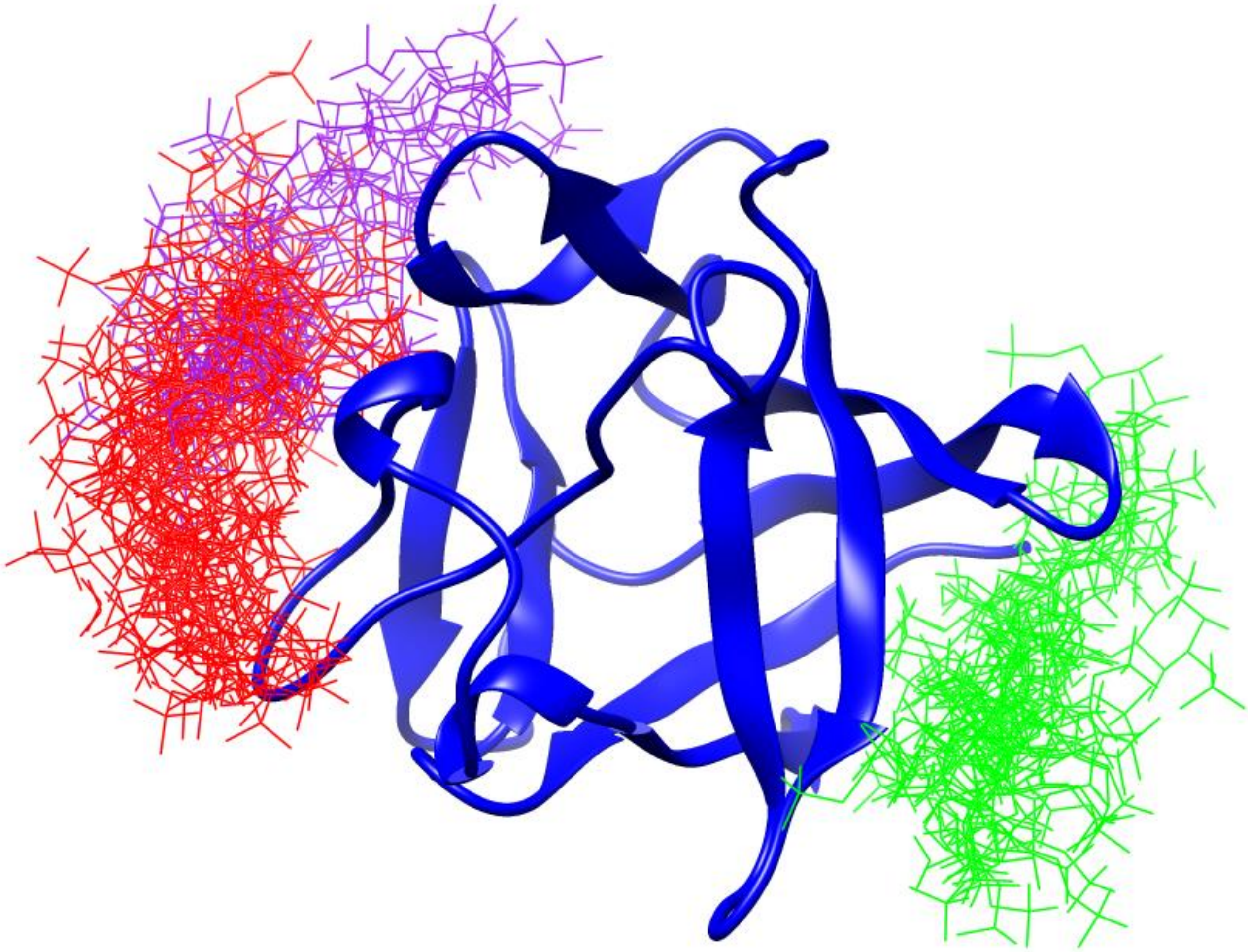

Supplementary Figure 3. Docking results for FGF1-HE dp5 obtained with AD3. FGF1 is shown in blue cartoon representation in complex with clusters of HE dp5 (cluster 1 in red, cluster in 2 green and cluster 3 in purple). Clusters 1 and 3 occupy the experimentally observed binding region, while cluster 2 is located at the opposite site of FGF1.

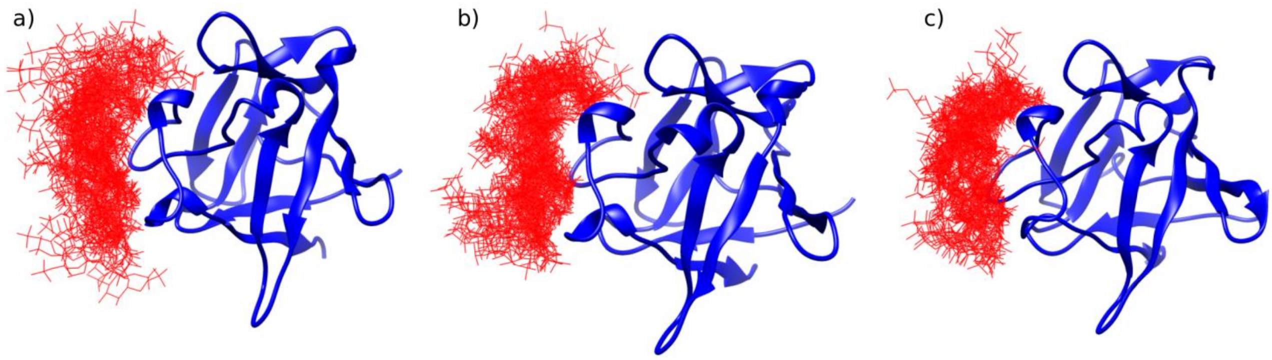

Supplementary Figure 4. Clusters of HE dp5 docking solutions (in red) obtained with AD3 are clustered around the experimentally observed binding site of FGF1 (in blue cartoon representation). Selected clusters are shown for (a) receptor conformation 5, (b) receptor conformation 6, and (c) receptor conformation 10.

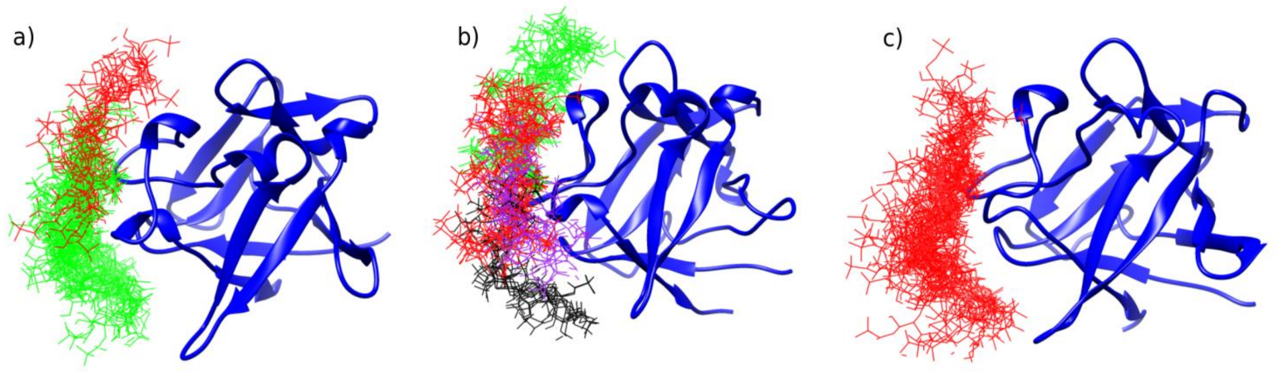

Supplementary Figure 5. Clusters of HE dp5* docking solutions on FGF1 obtained with AD3. (a) Docking to receptor conformation 6, which yields two clusters (1 in red and 2 in green). In this case, cluster 2 is extended further from the FGF1 HE binding loop. (b) Docking to receptor conformation 8 yields four clusters (1 in red), 2 in green, 3 in purple and 4 in black). Cluster 1 is positioned around the binding site with most of the sugar units interaction with the FGF1 binding loop, while the other clusters show only 2-3 sugar units interacting. (c) Docking to receptor conformation 10 yields only one cluster (red), which elongates on the surface of the protein.

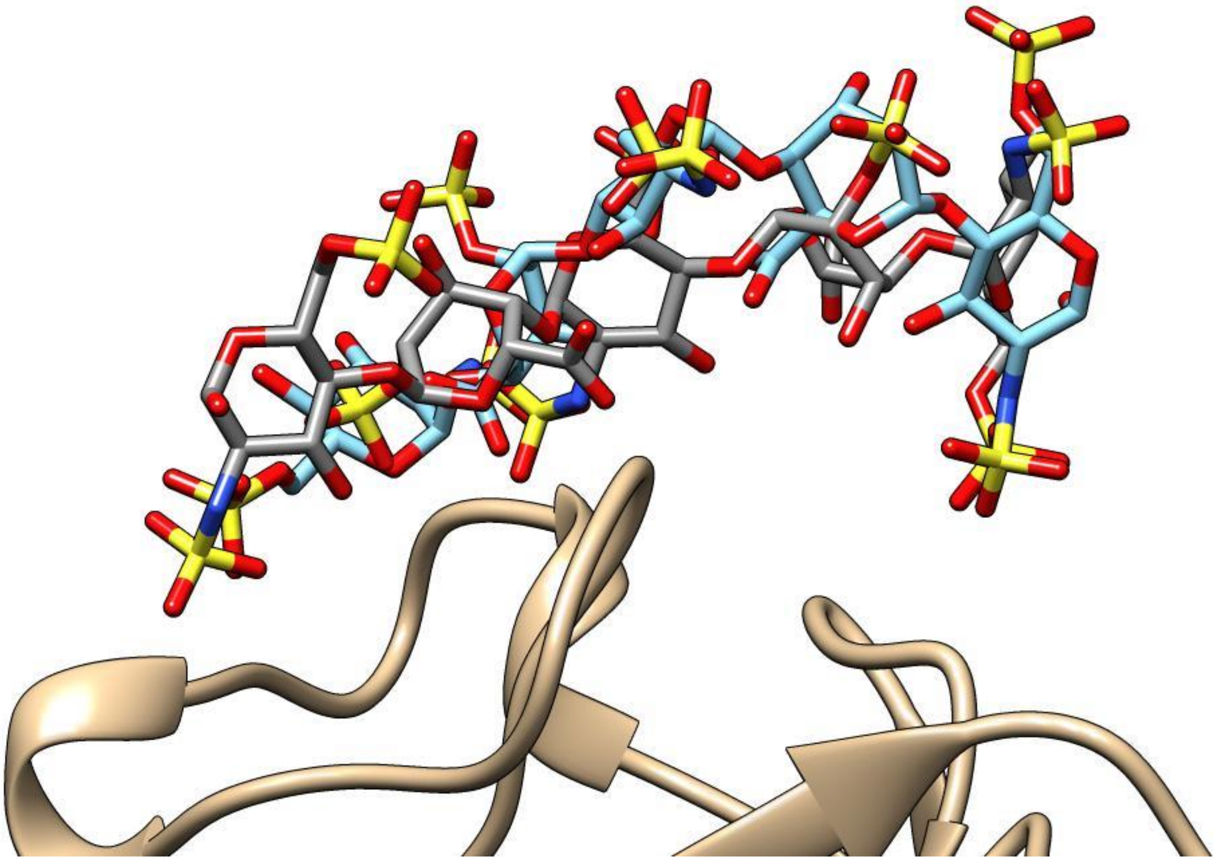

Supplementary Figure 6. Superimposed binding poses of HE dp5 (C atoms in grey) and HE dp5* (C atoms in blue) with receptor conformation 5 (beige cartoon). The two ligands occupy the same site with opposite polarity. HE dp5 binds to the protein loop via its non-reducing end, while HE dp5* does it through its reducing end.

FGF1 receptor conformation	HE1	HE2	HE3	HE4	HE5
1	-49.5	-62.0	-48.4	-58.5	-53.5
	-70.2	n.a.	-45.9	n.a.	n.a.
2	-87.8	-63.8	-74.6	-60.9	-59.4
	n.a.	n.a.	-84.3	n.a.	n.a.
3	-82.1	-66.8	-77.0	-60.2	-47.0
	n.a.	n.a.	n.a.	n.a.	-72.5
4	-58.9	-38.5	-57.6	-58.7	-47.9
	n.a.	n.a.	n.a.	-36.7	-65.3
	n.a.	n.a.	n.a.	n.a.	-27.9
5	-72.1	-72.8	-90.2	-55.0	-56.1
	n.a.	n.a.	-71.6	n.a.	n.a.
	n.a.	n.a.	-73.1	n.a.	n.a.
6	-96.7	62.6	-88.6	-46.7	-60.0
7	-71.4	-73.4	-93.7	-49.4	-71.1
8	-81.6	-73.1	-71.9	-45.3	-58.8
	n.a.	-69.2	n.a.	n.a.	n.a.
	n.a.	-59.0	n.a.	n.a.	n.a.
9	-84.4	-63.0	-60.1	-46.1	-40.5
	-13.4	n.a.	-74.1	n.a.	n.a.
10	-106.6	-52.8	-92.6	-53.4	-79.9
	-35.1	-63.8	n.a.	n.a.	-66.2

Supplementary Table 3. MM-GBSA ΔG binding (kcal/mol) for HE dp6 derivatives obtained from the MD simulations of AD3 docking solutions. Each number in the table corresponds to an analyzed cluster representative.

	Rank 1	Rank 2	Rank 3	Rank 4	Rank 5	Appearance ranking (Points)
HE1	7	1	2	-	-	45
HE2	-	4	3	2	1	30
HE3	2	5	1	1	1	36
HE4	-	-	2	2	6	16
HE5	1	-	2	5	2	23

Supplementary Table 4. Times that each ligand HE1-HE5 scored in each ranking position 1 to 5 (based on MM-GBSA ΔG binding energies for HE dp6 derivatives displayed in Supplementary Table 2). For appearance ranking, rank 1 scores 5 points, rank 2 scores 4, and thus successively to rank 5 which scores 1 point.

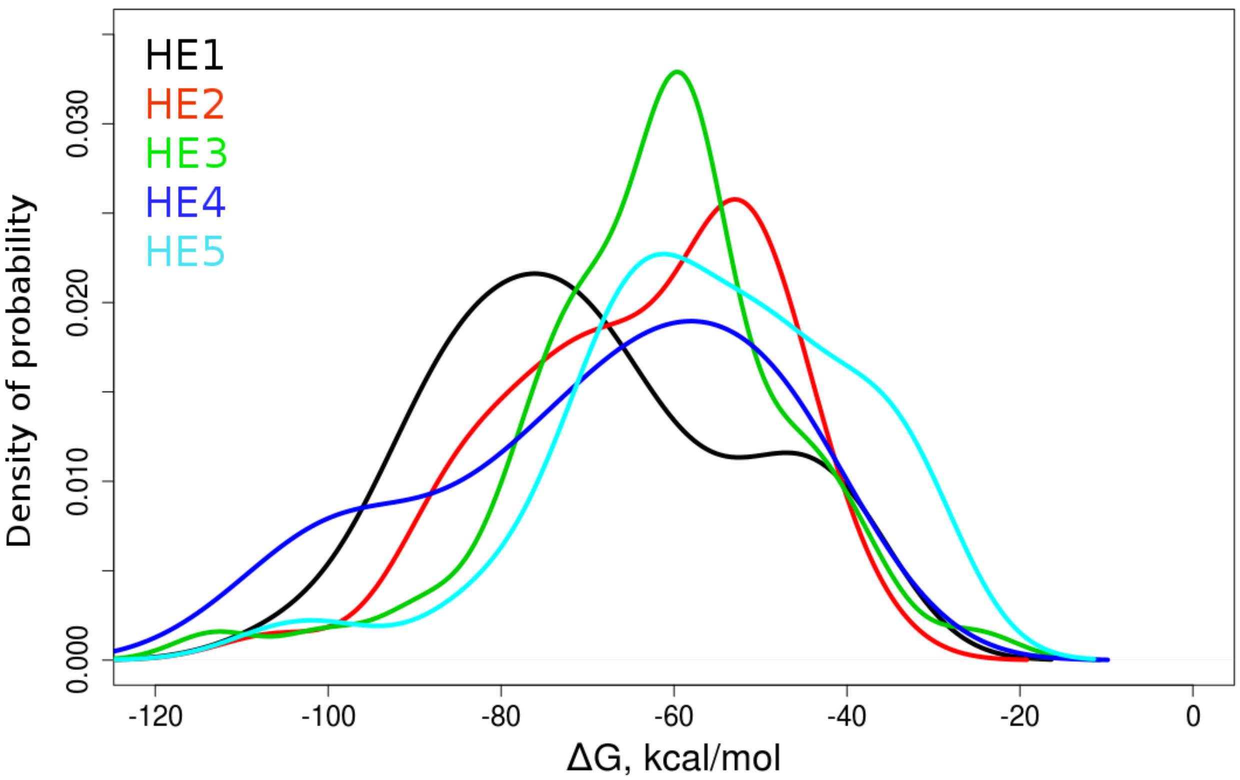

Supplementary Figure 7. Density of probability for free energy of binding (ΔG) for HE dp6 derivatives from the simulations of docking solutions obtained by DMD.

	H-bonds AD3						H-bonds DMD				
	HE1	HE2	HE3	HE4	HE5	HE1	HE2	HE3	HE4	HE5	
Crystal	0.40	0.26	0.38	0.13	0.29	0.24	0.23	0.27	0.17	0.38	
HE1		0.64	0.26	0.33	0.53		0.62	0.47	0.89	0.60	
HE2			0.35	0.43	0.71			0.64	0.63	0.59	
HE3				0.36	0.32				0.45	0.56	
HE4					0.33					0.49	
HE5											
							Ele DMD				
	HE1	HE2	HE3	HE4	HE5	HE1	HE2	HE3	HE4	HE5	
Crystal	0.45	0.50	0.47	0.51	0.47	0.45	0.36	0.47	0.36	0.50	
HE1		0.82	0.36	0.59	0.76	0.38		0.75	0.75	0.94	0.71
HE2			0.46	0.59	0.73	0.13			0.77	0.69	0.72
HE3				0.60	0.43	0.05				0.75	0.65
HE4					0.49	0.10					0.55
HE5						0.53					
	HE-dp6**						VdW DMD				
	0.03	HE2	HE3	HE4	HE5	HE-dp6**	HE1	HE2	HE3	HE4	HE5
Crystal	0.12	0.28	0.47	0.36	0.26	-0.01	0.40	0.38	0.57	0.53	0.37
HE1	-0.09	0.79	0.39	0.70	0.74	0.21		0.79	0.75	0.93	0.75
HE2	-0.12		0.36	0.68	0.71	0.10			0.82	0.71	0.83
HE3	-0.06			0.55	0.30	0.02				0.80	0.66
HE4	0.18				0.69	0.21					0.61
HE5	Ele AD3					0.29					
	HE-dp6**										
	0.14										

Supplementary Table 5. Pearson correlation between the corresponding datasets of residue pairwise interactions obtained from the crystal structure and AD3/DMD docking procedures for HE dp6 derivatives. HE-dp6** denotes the fully desulfated HE.

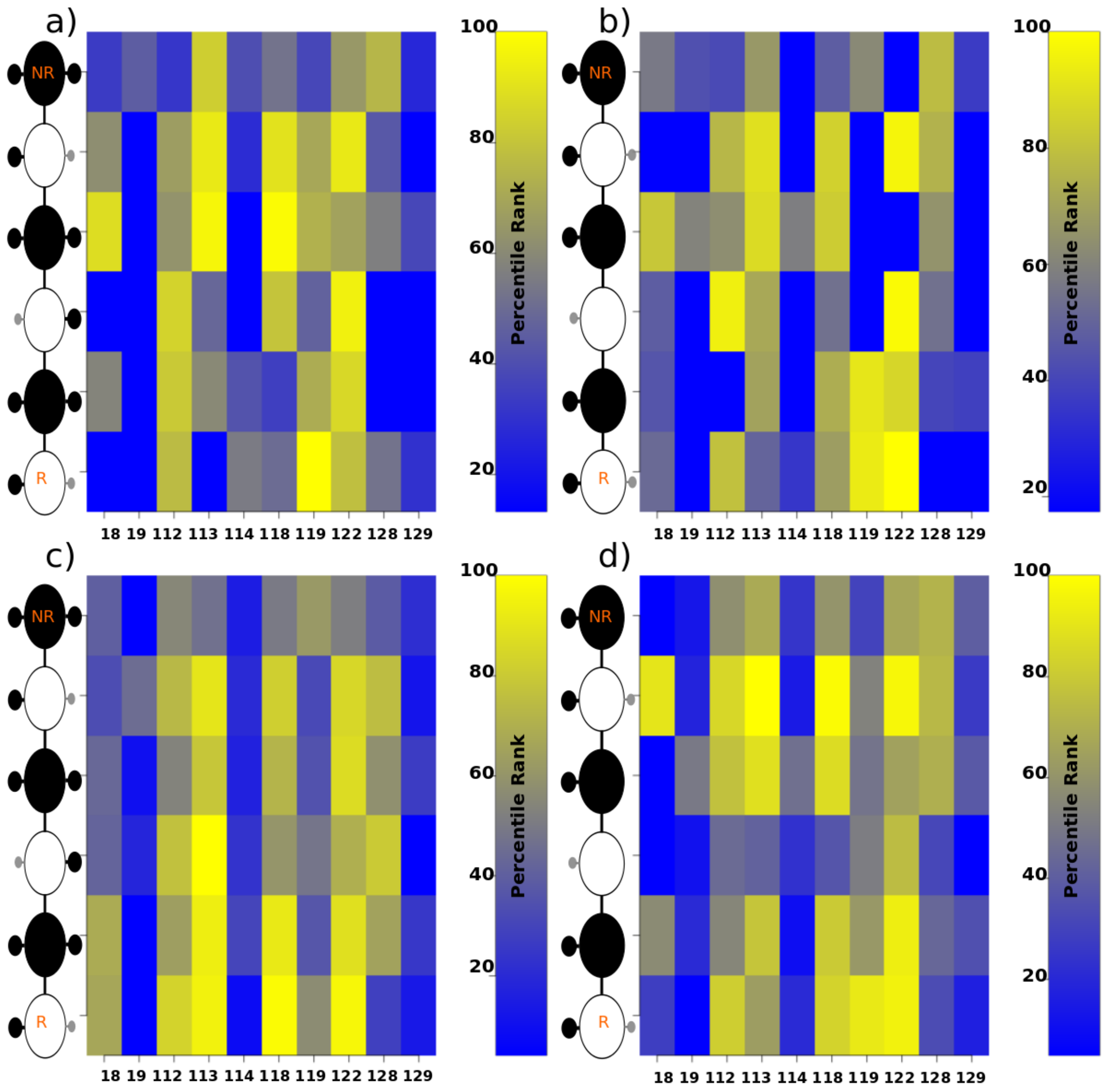

Supplementary Figure 8. H-bonding pattern obtained for HE1 (a) and HE2 (b) by AD3 ; H-bonding pattern obtained for HE1 (c) and HE2 (d) by DMD. Protein residue numbers and GAG residues are indicated in x- and y-axis, respectively. NR and R indicate the non-reducing and reducing ends, respectively.

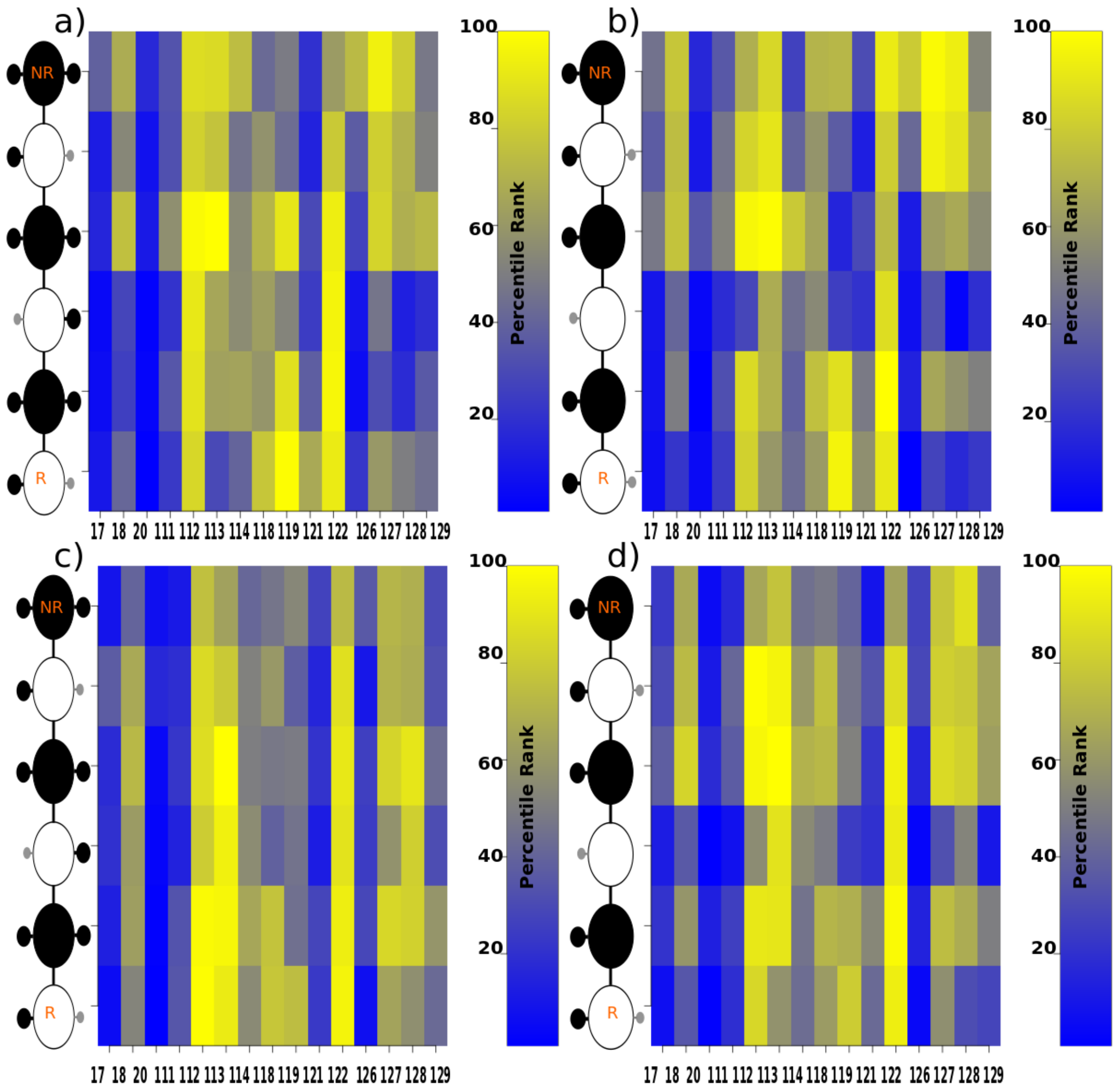

Supplementary Figure 9. Vdw interactions obtained for HE1 (a) and HE2 (b) by AD3; vdw interactions obtained for HE1 (c) and HE2 (d) by DMD. Protein residue numbers and GAG residues are indicated in x- and y-axis, respectively. NR and R indicate the non-reducing and reducing ends, respectively.

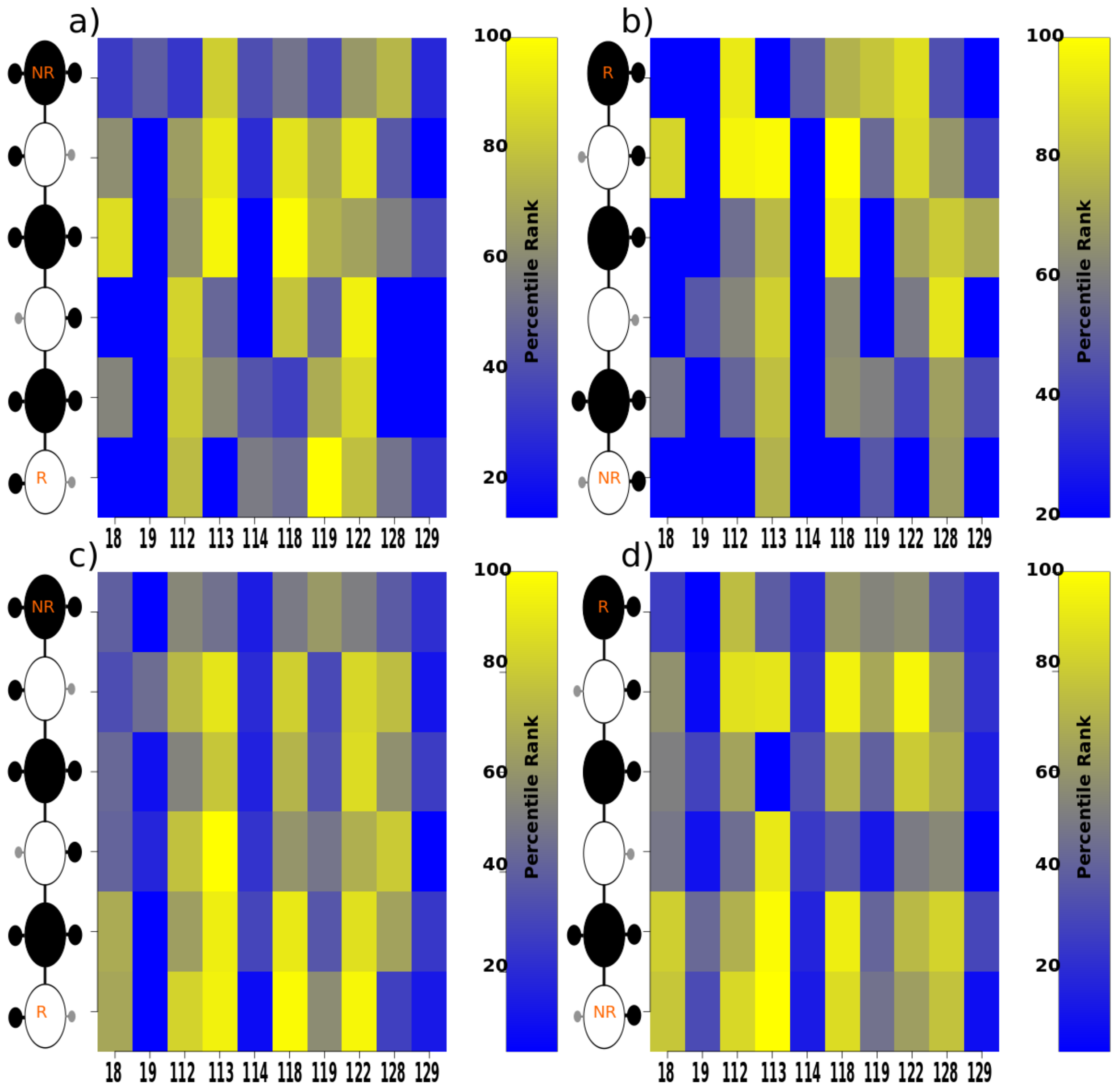

Supplementary Figure 10. H-bonding pattern obtained for HE1 (a) and HE3 (b) by AD3; H-bonding pattern obtained for HE1 (c) and HE3 (d) by DMD. Protein residue numbers and GAG residues are indicated in x- and y-axis, respectively. NR and R indicate the non-reducing and reducing ends, respectively.

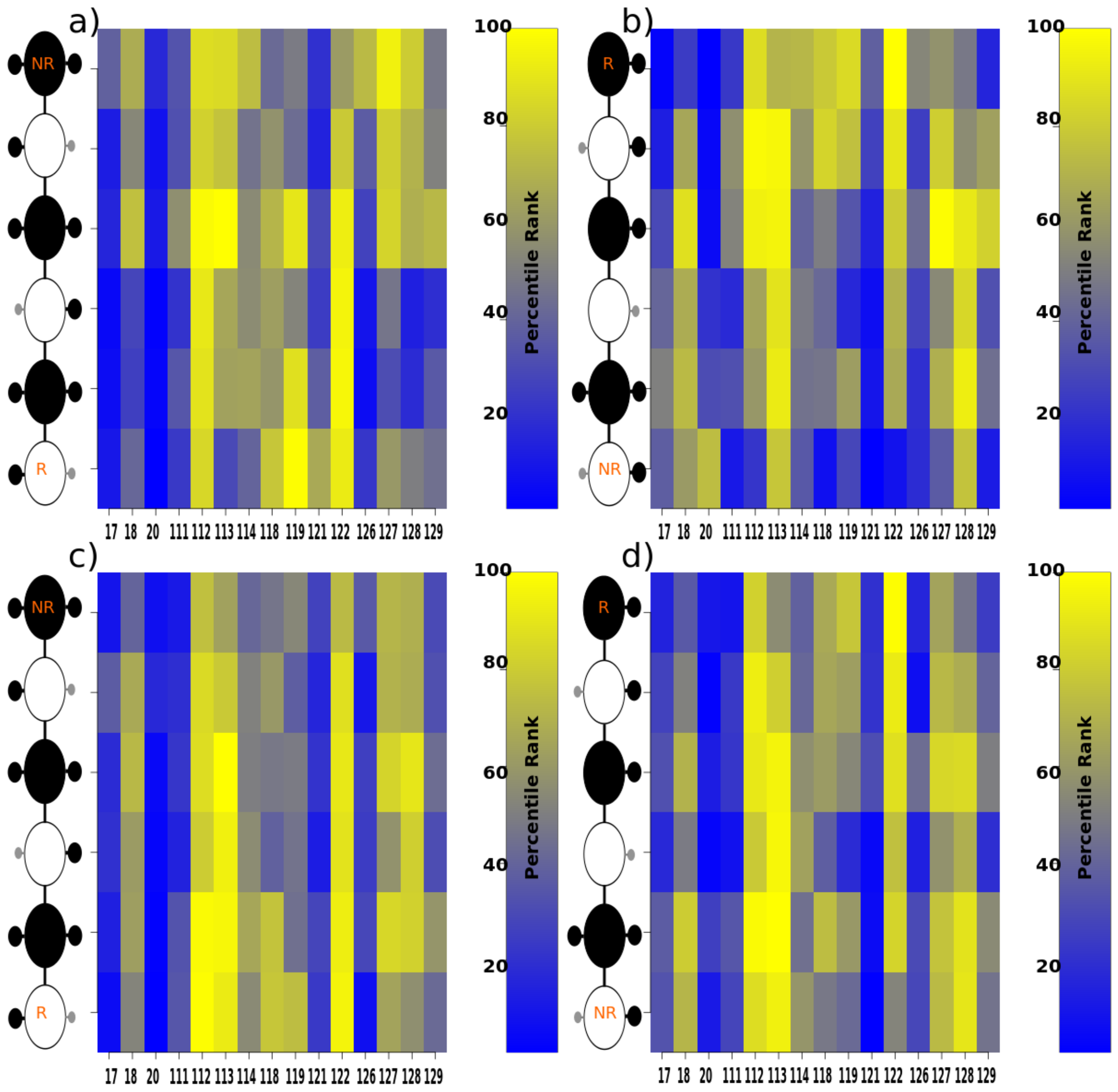

Supplementary Figure 11. Vdw interactions obtained for HE1 (a) and HE3 (b) by AD3; vdw interactions obtained for HE1 (c) and HE3 (d) by DMD. Protein residue numbers and GAG residues are indicated in x- and y-axis, respectively. NR and R indicate the non-reducing and reducing ends, respectively.

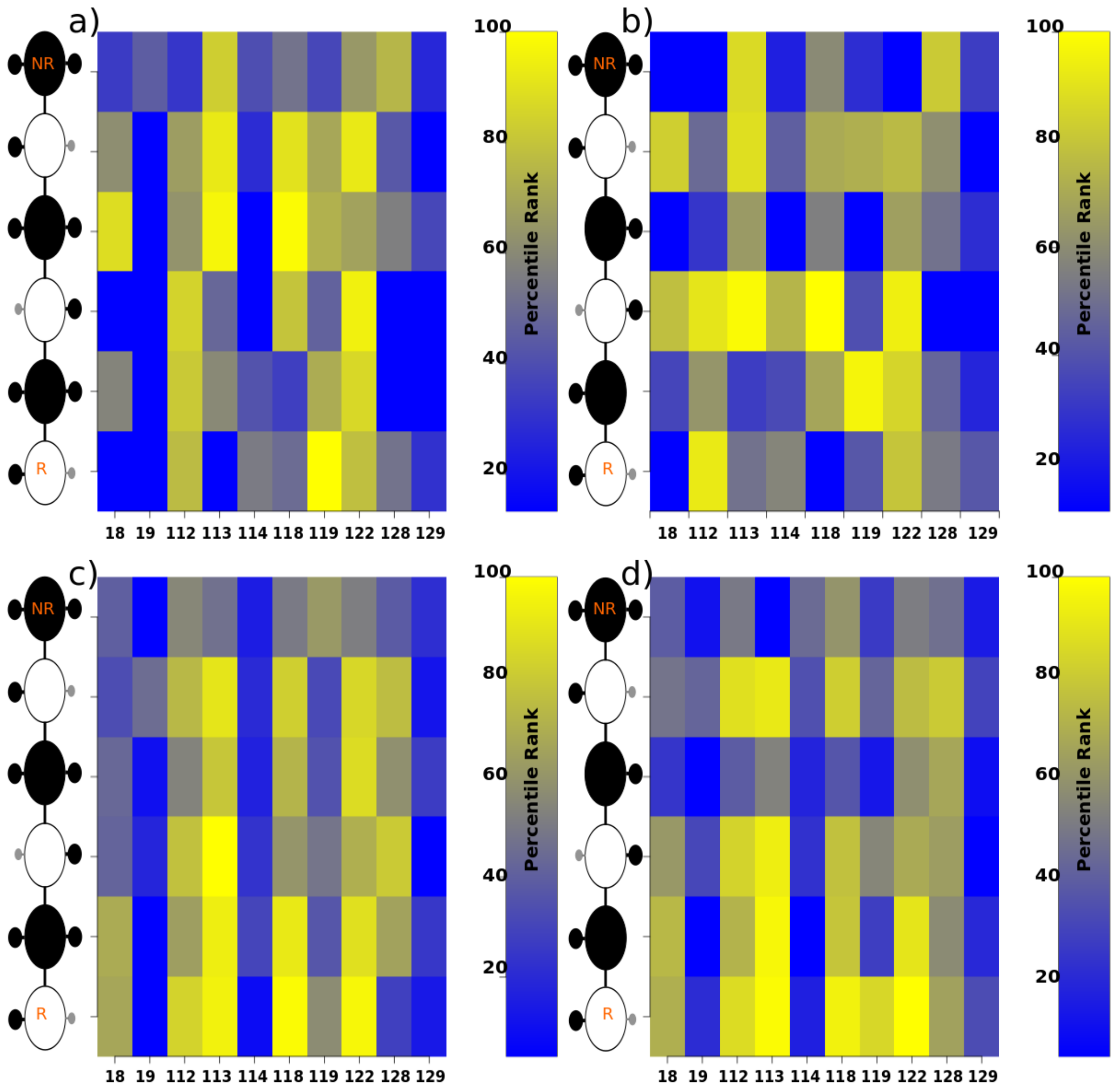

Supplementary Figure 12. H-bonding pattern obtained for HE1 (a) and HE4 (b) by AD3; H-bonding pattern obtained for HE1 (c) and HE4 (d) by DMD. Protein residue numbers and GAG residues are indicated in x- and y-axis, respectively. NR and R indicate the non-reducing and reducing ends, respectively.

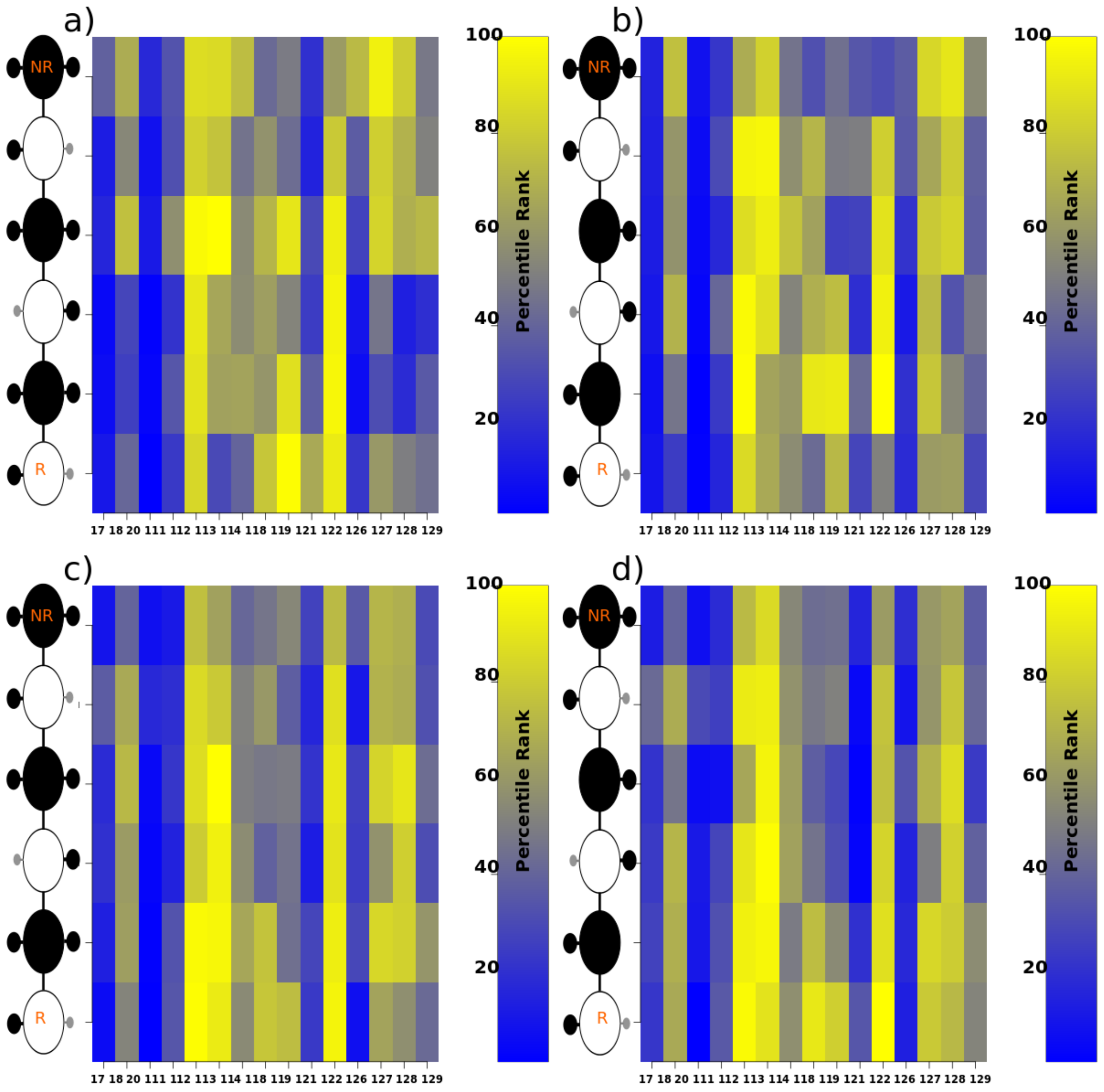

Supplementary Figure 13. Vdw interactions obtained for HE1 (a) and HE4 (b) by AD3; vdw interactions obtained for HE1 (c) and HE4 (d) by DMD. Protein residue numbers and GAG residues are indicated in x- and y-axis, respectively. NR and R indicate the non-reducing and reducing ends, respectively.

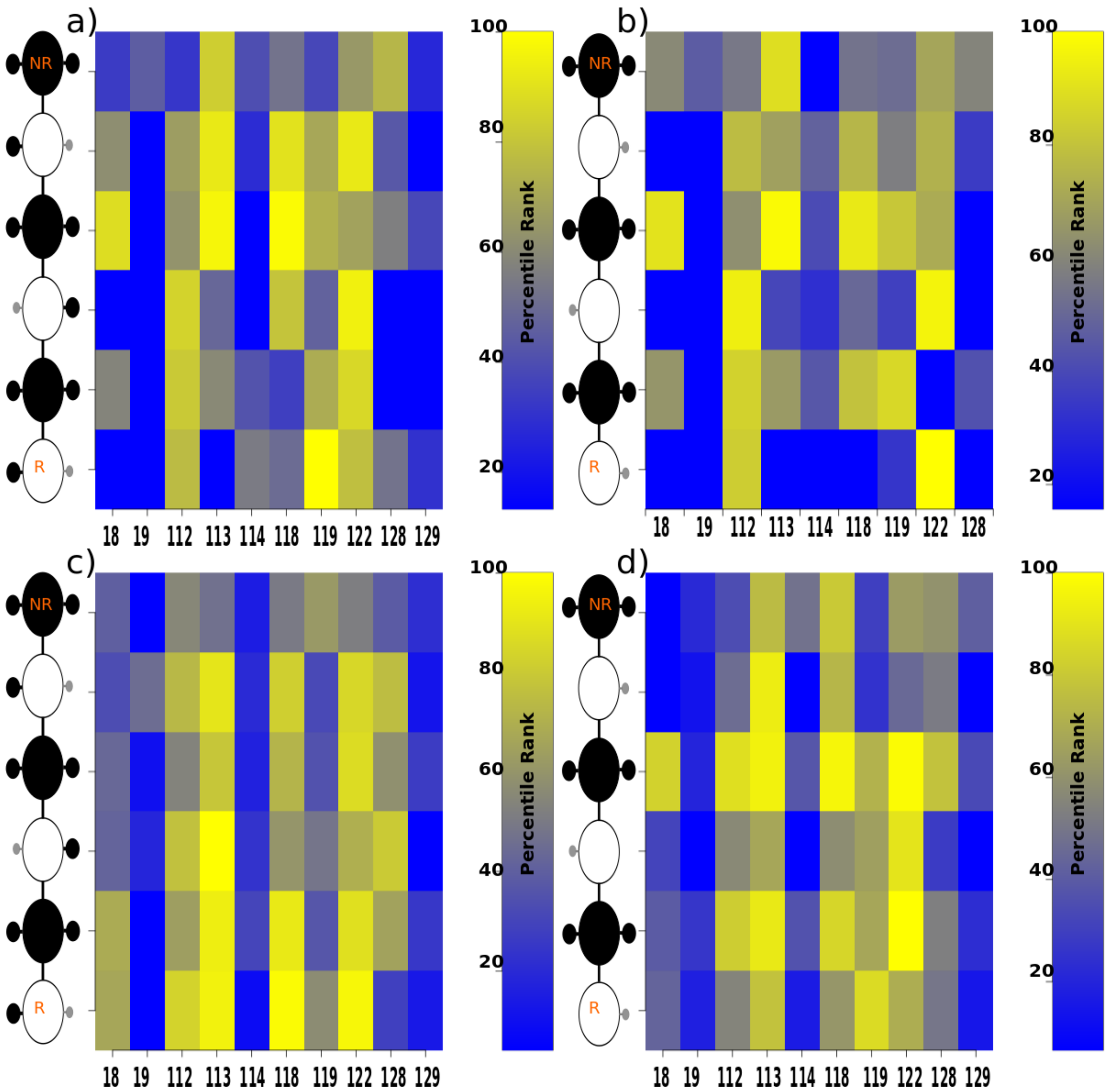

Supplementary Figure 14. H-bonding pattern obtained for HE1 (a) and HE5 (b) by AD3; H-bonding pattern obtained for HE1 (c) and HE5 (d) by DMD. Protein residue numbers and GAG residues are indicated in x- and y-axis, respectively. NR and R indicate the non-reducing and reducing ends, respectively.

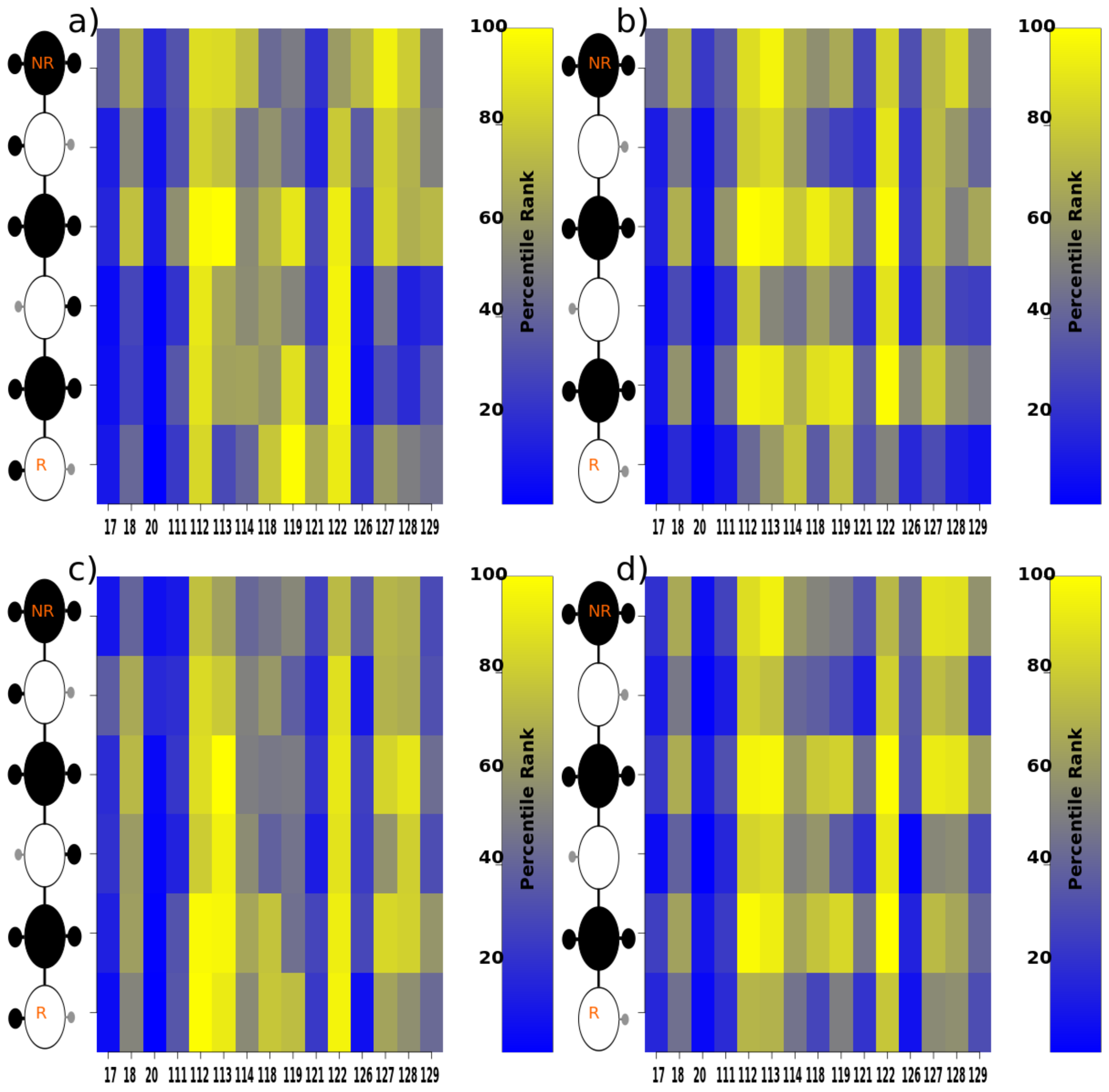

Supplementary Figure 15. Vdw interactions obtained for HE1 (a) and HE5 (b) by AD3; H-bonding pattern obtained for HE1 (c) and HE5 (d) by DMD. Protein residue numbers and GAG residues are indicated in x- and y-axis, respectively. NR and R indicate the non-reducing and reducing ends, respectively.

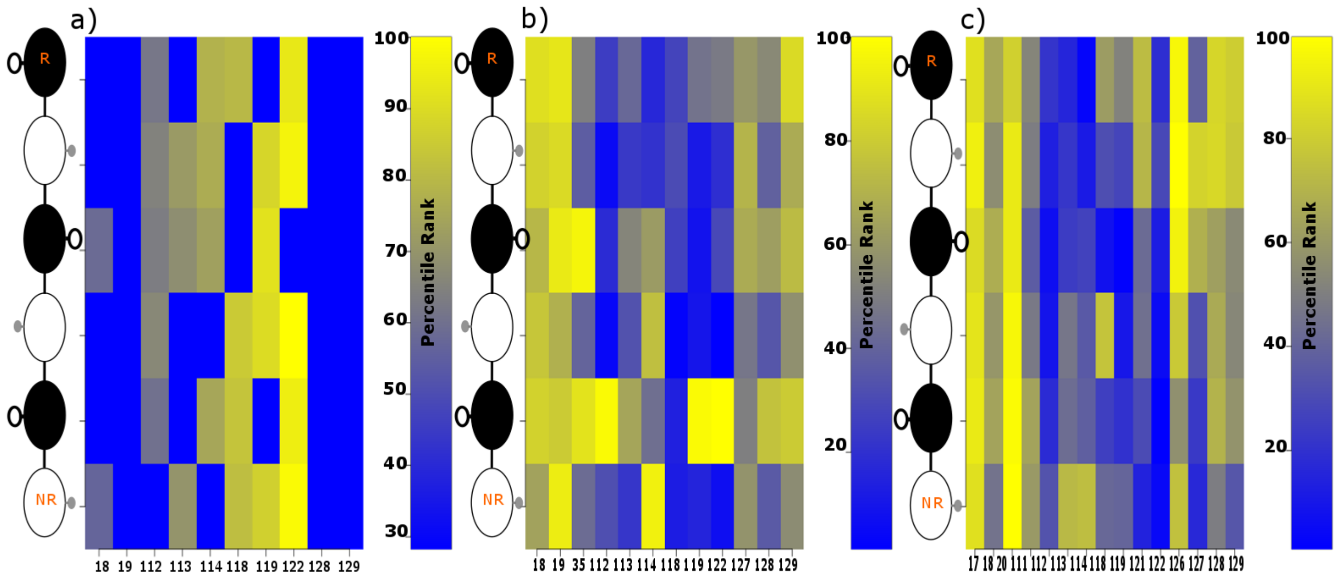

Supplementary Figure 16. H-bonding pattern (a), Ele (b) and vdW(c) interactions obtained by AD3 for HE-dp6**. Protein residue numbers and GAG residues are indicated in x- and y-axis, respectively. NR and R indicate the non-reducing and reducing ends, respectively.
